# Tonic GABA_A_ Receptor-Mediated Currents of Human Cortical GABAergic Interneurons Vary Amongst Cell Types

**DOI:** 10.1523/JNEUROSCI.0175-21.2021

**Published:** 2021-11-24

**Authors:** Martin Field, Istvan P. Lukacs, Emily Hunter, Richard Stacey, Puneet Plaha, Laurent Livermore, Olaf Ansorge, Peter Somogyi

**Affiliations:** ^1^Department of Pharmacology, University of Oxford, Oxford OX1 3QT, United Kingdom; ^2^Department of Neurosurgery, John Radcliffe Hospital, Oxford University Hospitals NHS Foundation Trust, Oxford OX3 9DU, United Kingdom; ^3^Nuffield Department of Clinical Neurosciences, University of Oxford, Oxford OX3 9DU, United Kingdom

**Keywords:** GABA_A_, human cortex, inhibition, interneuron, seizures, tonic

## Abstract

Persistent anion conductances through GABA_A_ receptors (GABA_A_Rs) are important modulators of neuronal excitability. However, it is currently unknown how the amplitudes of these currents vary among different cell types in the human neocortex, particularly among diverse GABAergic interneurons. We have recorded 101 interneurons in and near layer 1 from cortical tissue surgically resected from both male and female patients, visualized 84 of them and measured tonic GABA_A_R currents in 48 cells with an intracellular [Cl^–^] of 65 mm and in the presence of 5 μm GABA. We compare these tonic currents among five groups of interneurons divided by firing properties and four types of interneuron defined by axonal distributions; rosehip, neurogliaform, stalked-bouton, layer 2–3 innervating and a pool of other cells. Interestingly, the rosehip cell, a type of interneuron only described thus far in human tissue, and layer 2–3 innervating cells exhibit larger tonic currents than other layer 1 interneurons, such as neurogliaform and stalked-bouton cells; the latter two groups showing no difference. The positive allosteric modulators of GABA_A_Rs allopregnanolone and DS2 also induced larger current shifts in the rosehip and layer 2–3 innervating cells, consistent with higher expression of the δ subunit of the GABA_A_R in these neurons. We have also examined how patient parameters, such as age, seizures, type of cancer and anticonvulsant treatment may alter tonic inhibitory currents in human neurons. The cell type-specific differences in tonic inhibitory currents could potentially be used to selectively modulate cortical circuitry.

**SIGNIFICANCE STATEMENT** Tonic currents through GABA_A_ receptors (GABA_A_Rs) are a potential therapeutic target for a number of neurologic and psychiatric conditions. Here, we show that these currents in human cerebral cortical GABAergic neurons display cell type-specific differences in their amplitudes which implies differential modulation of their excitability. Additionally, we examine whether the amplitudes of the tonic currents measured in our study show any differences between patient populations, finding some evidence that age, seizures, type of cancer, and anticonvulsant treatment may alter tonic inhibition in human tissue. These results advance our understanding of how pathology affects neuronal excitability and could potentially be used to selectively modulate cortical circuitry.

## Introduction

The signaling mediated by anion conductances through GABA_A_ receptors (GABA_A_Rs) takes two forms: phasic and tonic (Farrant and Nusser, 2005; Farrant and Kaila, 2007). Phasic signaling is mediated by GABA_A_Rs concentrated in the synaptic junctions briefly opening in response to large but transient elevations in the concentration of GABA in the synaptic cleft (Mody et al., 1994; Mozrzymas et al., 2003; Overstreet et al., 2003; Mozrzymas, 2004), resulting from vesicular GABA release. Conversely, tonic inhibition through GABA_A_Rs involves a persistent conductance that is usually generated in response to low concentrations of GABA (Kaneda et al., 1995; Brickley et al., 1996; Tia et al., 1996; Wall and Usowicz, 1997; Farrant and Nusser, 2005; Farrant and Kaila, 2007) in the extracellular space.

These two modes of signaling differ in the subunit compositions of the GABA_A_Rs underlying them (Nusser et al., 1998; Mody, 2001; Farrant and Nusser, 2005; Farrant and Kaila, 2007; Glykys and Mody, 2007; Belelli et al., 2009; Brickley and Mody, 2012), with phasic being primarily mediated by receptors composed of α(1–3, or 5) subunits along with two β and γ2 (Essrich et al., 1998; Chua and Chebib, 2017) subunits, while receptors mediating tonic inhibition include α(4–6), β and either δ (for α4 or 6), or γ (for α5) subunits (Glykys et al., 2008; Belelli et al., 2009; Brickley and Mody, 2012). However, these subunit combinations are only tendencies, as tonic currents mediated by α1 and α2 (Glykys et al., 2007; Yamada et al., 2007; Prokic et al., 2015; Durkin et al., 2018) have been reported. Indeed, the α1, α2, and β3 subunits content in the extrasynaptic plasma membrane far exceeds that in synaptic junctions (Kasugai et al., 2010).

Receptors underlying tonic inhibition and their associated conductances are expressed across the brain (Lee and Maguire, 2014). Of the GABA_A_R subunits considered most relevant to tonic inhibition (α4–6 and δ), the α6 shows limited distribution, being found mainly in cerebellar granule cells (Pirker et al., 2000), while α4–5 and δ show expression across most brain regions (Pirker et al., 2000; Sieghart and Sperk, 2002). Furthermore, tonic currents of varying amplitudes have been reported for numerous glutamatergic principal cells and GABAergic interneurons (Lee and Maguire, 2014). Interestingly, the tonic currents of interneurons appear to be strong regulators of network activity in the neocortex, with the δ subunit-preferring agonist 4,5,6,7-tetrahydroisoxazolo[5,4-c]pyridin-3-ol (THIP) increasing excitatory signaling and reducing inhibitory neurotransmission (Krook-Magnuson and Huntsman, 2005; Drasbek and Jensen, 2006).

The increasing resolution of cell types in the neocortex (Hodge et al., 2019), raises the question of to what degree are neurons differentially regulated by tonic GABA_A_R conductances. While differences in the tonic currents of different pyramidal cells of the neocortex of non-human mammals have been well studied (Yamada et al., 2007; Jang et al., 2013), which cortical interneurons are regulated by tonic GABA_A_R currents remains to be addressed. The need for understanding the modulation of different cell types by tonic currents is important in the human cortex, where numerous inhibitory cell types have been revealed by anatomic, transcriptomic, and physiological methods (Hawrylycz et al., 2012; Varga et al., 2015; Boldog et al., 2018; Hodge et al., 2019). Although human cortical interneurons and principal cells are already known to express tonic GABA_A_R conductances (Scimemi et al., 2006), a finer cell type-specific analysis may reveal differences that provide an avenue for a selective modulation of cortical circuitry.

In this study, human brain transcriptomic data (Hawrylycz et al., 2012; Hodge et al., 2019; Bakken et al., 2020; BRAIN Initiative Cell Census Network, 2020) was first analyzed to identify differences in the expression of GABA_A_R subunits among human layer 1 cortical interneurons. Single cell patch-clamp was then used to measure tonic GABA_A_R-mediated currents in different types of visualized layer 1 human cortical interneurons from surgical samples, in an attempt to investigate to what degree different cell types display differences in the amplitude of the tonic currents.

## Materials and Methods

### Tissue collection and slice preparation

Samples of human cortical tissue were obtained from consenting patients undergoing surgical treatment at the John Radcliffe Hospital (Oxford) for a variety of cancers of the brain (Table 1), in addition to a single patient whose cortical tissue was removed as part of treatment for temporal lobe epilepsy. Tissue from both male and female patients was used in this study (Table 1). In all cases, ethical approval was given through the Oxford Brain Bank, and informed patient consent was obtained by individuals not involved in the study. Only tissue being removed during surgery, which would have otherwise been discarded, was taken for this study. The samples represented so-called “access tissue,” that is, cortical tissue necessarily removed during surgery to gain access to the diseased part of the brain and deemed as close to normal cortex as is possible in these circumstances.

**Table 1.** Patient parameters for all samples from which at least one tonic current measurement was made

Case	Age	Sex	Pathology	Cortical area	Perioperative treatment	Seizures	Onset of seizures	Anticonvulsant medication	Dexamethasone treatment
1	50	M	Temporal lobe epilepsy	Right inferior temporal gyrus	Remifentanyl, propofol, rocuronium co-amoxiclav, dexamethasone	Yes, 5 years absence seizures and 3 years tonic/clonic	5 years	Carbamazepine 800 mg morning 600 mg evening, clobazam 20 mg od, lamotrigine 300 mg od, levetiracetam 2 g bd	None
2	36	F	Germinoma	Right frontal superior gyrus	Propofol, remifentanyl, atracurium, co-amoxiclav, dexamethasone, MgSo, hydrocortisone	No	NA	None	None
3	43	M	Oligodendroglioma	Right inferior temporal gyrus	propofol, remifentanyl, dexamethasone, atracurium, co-amoxiclav	Yes, focal	5 years	1000 mg A.M. and 1500 mg P.M. levetiracetam (note, no reported seizures in the 3 years preceding the surgery)	16 mg dexamethasone at time of surgery
4	48	F	Metastatic adenocarcinoma	Right superior frontal gyrus	Not available	No	NA	None	Dexamethasone: 4 mg/day for 6 d prior and 16 mg at time of surgery
5	64	F	Glioblastoma	Right inferior temporal gyrus	Remifentanyl, propofol, rocuronium, co-amoxiclav	No	NA	None	None
6	61	M	Glioblastoma	Right middle temporal gyrus	Remifentanyl, propofol, atracurium, metaraminol,coamoxiclav,	No	NA	Levetiracetam 500 mg bd (prophylactic)	16 mg dexamethasone at time of surgery
7	57	M	Glioblastoma	Right frontal middle gyrus	Not available	No	NA	Levetiracetam 500 mg bd (prophylactic)	16 mg dexamethasone at time of surgery
8	56	F	Meningioma	Right middle temporal gyrus	Propofol, remifentanyl, dexamethasone, atracurium, co-amoxiclav	Yes, generalized tonic/clonic	2 months	Levetiracetam 500 mg bd	None
9	61	M	Glioblastoma	Left middle temporal gyrus	Remifentanyl, propofol, atracurium, co-amoxiclav	Yes, focal	15 d	Levetiracetam 500 mg bd	Dexamethasone: 8 mg/d for 15 d prior and on the day of surgery
10	54	M	Glioblastoma	Right inferior temporal gyrus	Propofol, remifentanyl, dexamethasone, atracurium, co-amoxiclav	No	NA	Levetiracetam 250 mg bd (prophylactic)	16 mg dexamethasone at time of surgery
11	38	F	Anaplastic astrocytoma	Right inferior temporal gyrus	Remifentanyl, propofol, vecuronium, dexamethasone, co-amoxiclav, atracurium	Yes, focal and secondary generalized	10 d	Levetiracetam 1 g bd	Dexamethasone: 8 mg/d for 10 d prior and 16 mg on the day of surgery
12	50	F	Glioblastoma	Left inferior temporal gyrus	Remifentanyl, propofol, rocuronium, ceftriaxone	Yes, generalized tonic/clonic	2 months	Levetiracetam 500 mg bd	None
13	72	M	Metastatic carcinoma	Right middle frontal gyrus	Remifentanyl, propofol, rocuronium, co-amoxiclav	No	NA	None	None
14	64	M	Dysembryoplastic neuroepithelial tumor	Right inferior parietal lobule	Propofol, remifentanyl, dexamethasone, atracurium, co-amoxiclav	No	NA	None	16 mg dexamethasone at time of surgery
15	60	M	Anaplastic astrocytoma	Right inferior temporal gyrus	Propofol, remifentanyl, dexamethasone, atracurium, co-amoxiclav	Yes, focal	6 years	Levetiracetam 500 mg bd (note, patient had no reported seizures in the 4 years preceding the surgery)	None
16	42	M	Glioblastoma	Left superior parietal lobule	Remifentanyl, propofol, atracurium, co-amoxiclav	No	NA	None	Dexamethasone: 8 mg/d for 10 d prior and 16 mg at time of surgery
17	69	F	Glioblastoma	Right superior frontal gyrus	Remifentanyl, propofol, atracurium, co-amoxiclav	No	NA	None	Dexamethasone: 4 mg/d for 2 weeks prior and 16 mg at time of surgery
18	56	F	Oligodendroglioma	Left superior frontal gyrus	Remifentanyl, propofol, vecuronium, dexamethasone, co-amoxiclav, atracurium	No	NA	None	None
19	45	F	Metastatic adenocarcinoma	Right superior occipital gyrus	Remifentanyl, propofol, atracurium, co-amoxiclav	No	NA	None	Dexamethasone: 4 mg/d for 3 weeks prior and 16 mg at time of surgery
20	53	M	Glioblastoma	Left middle temporal gyrus	Remifentanyl, propofol, atracurium, metaraminol, co-amoxiclav	No	NA	Levetiracetam 750 mg bd	None
21	50	M	Glioblastoma	Right inferior frontal gyrus	Remifentanyl, propofol, rocuronium, co-amoxiclav	No	NA	None	Dexamethasone: 8 mg/d for 3 weeks prior and 16 mg at time of surgery

od - once a day, bd - twice a day, NA - not applicable, M - male, F - female.

The tissue was immediately immersed and trimmed in ice-cold cutting artificial cerebrospinal fluid (ACSF; 2.5 mm KCl, 1.25 mm NaH_2_PO_4_, 30 mm NaHCO_3_, 20 mm HEPES, 25 mm glucose, 5 mm Na-ascorbate, 3 mm Na-pyruvate, 2 mm thiourea, 92 mm NMDG, 0.5 mm CaCl_2_, and 10 mm MgSO_4_, pH 7.3) saturated with carbogen (95% O_2_, 5% CO_2_). The tissue was then placed into a sealed container filled with ice cold cutting ACSF saturated with carbogen for transport to the laboratory for slicing (10–15 min).

The tissue was then sliced into 350 μm thick slices on a vibratome (either a Leica VT1200, or a Zeiss Hyrax v50). Slicing was conducted in 4°C cutting ACSF saturated with carbogen. The resulting slices were allowed to recover at 37°C for 10–15 min before being cooled to room temperature and transferred to a holding chamber containing storing ACSF (2.5 mm KCl, 1.25 mm NaH_2_PO_4_, 30 mm NaHCO_3_, 20 mm HEPES, 25 mm glucose, 5 mm Na-ascorbate, 3 mm Na-pyruvate, 2 mm thiourea, 92 mm NaCl, 2 mm CaCl_2_, and 2 mm MgSO_4_, pH 7.3). Slices were subsequently maintained submerged in this storing media at room temperature (∼21°C) until being used for electrophysiological recordings. The storing chamber was continuously bubbled with carbogen to ensure oxygenation of the tissue and to maintain the pH of the solution.

### Patch-clamp electrophysiology

Recordings took place 3–24 h following removal of the tissue from the patient. Slices were placed into the bath chamber of the recording rig and were submersed under continuous flow of recording ACSF (130 mm NaCl, 3.5 mm KCl, 1.5 mm NaH_2_PO_4_, 24 mm NaHCO_3_, 12.5 mm glucose, 1.5 mm MgSO_4_, and 3 mm CaCl_2_) saturated with carbogen and heated to achieve a bath temperature of ∼32–34°C. The slices were visualized using infrared differential interference contrast (DIC) microscopy. Cell bodies located in layer 1 (as judged by proximity to the pial surface, and by the absence of pyramidal cell somas) were targeted for whole-cell patching with electrodes of 3- to 5-MΩ resistance, filled with an internal solution (60 mm KCl, 80 mm K-gluconate, 10 mm HEPES, 5 mm EGTA, 2 mm MgCl_2_, 0.5 mm CaCl_2_, 4 mm Mg-ATP, 0.5 mm Na-GTP, and 0.2% biocytin, pH 7.2).

Recordings were made using either an EPC-10 triple amplifier in combination with Patchmaster software (HEKA), or with a Multiclamp 700B, connected to a Digidata 1550, in combination with pClamp (Molecular Devices). For current clamp recordings, cells were clamped within a range of −100–0 pA to maintain a membrane potential of approximately −60 mV. Current–voltage (IV) traces were generated by applying 20-pA current steps for 800 ms, across a total current range of at least the holding current minus 100 pA to rheobase plus 100 pA. Voltage responses to these current steps were recorded with a sample rate of 100 kHz.

For measurements of tonic GABA_A_R-mediated currents, a voltage-clamp of −60 mV was subsequently applied, in addition to a series resistance compensation of 80–85%. For such current recordings, a baseline period of at least 3 min was recorded before the application of NBQX (20 μm, Abcam), DL-2-amino-5-phosphonovaleric acid (APV; 50 μm, Santa Cruz Biotechnology), and γ-aminobutyric acid (GABA) (5 μm). The exogenous GABA was added in an attempt to standardize the ambient GABA concentration (Glykys and Mody, 2006; Bright and Smart, 2013), which may vary depending on numerous factors such as the length of time since tissue removal, and the depth of the cell within the slice. These drugs were allowed to wash in for a further 3 min before the application of (-)-bicuculline methochloride (50 μm, Tocris). Once the bicuculline was applied, the holding current was allowed to reach a steady-state before any attempt to wash-off the drug. For a subset of cells (*n* = 17), the initial current-clamp IV measurement was then repeated in the presence of bicuculline before wash-off. Voltage-step pulses (10 mV) were applied at 5 min intervals throughout the experiment to monitor access resistance and recording periods were discarded if a change larger than 25% was observed. Once the experiment was completed, for subsequent visualization of the cells, each slice was transferred to ice cold fixative [4% w/v paraformaldehyde, 15% v/v saturated picric acid, dissolved in 0.1 m phosphate buffer (PB), pH 7.2–7.4], and left overnight before being washed in 0.1 m PB. For measurements of the responses of the cells to allopregnanolone (0.5 μm, Tocris), DS2 (1 μm, Tocris), and MRK-016 (0.1 μm, Tocris), the drugs were applied and washed out sequentially before the application of bicuculline. All three of these drugs were applied in the presence of the 5 μm GABA. For all three drugs, 10 mm stocks were produced by dissolving them in DMSO to a concentration of 10 mm. An appropriate volume of these stock solutions was then added to a beaker of recording ACSF to produce a working solution with the indicated concentration.

### Clustering of cells based on firing

For the clustering of cells based on their firing properties, parameters were first extracted from current-clamp IV recordings using a custom MATLAB (version R2019b, MathWorks) script implementing the parameter extraction algorithm developed by the Allen Brain Institute (Gouwens et al., 2019). Overall, this method distils each IV trace into 13 total datasets (Gouwens et al., 2019), containing information about a variety of firing and membrane properties of each cell. Sparse principal component analysis (sPCA) was then used (conducted in R using the sparsepca package, version 0.1.2; Erichson et al., 2020) to reduce these 13 parameter sets down to a set of 21 variables (taking components accounting for up to 95% of the variance in each dataset). Clustering was then conducted using Gaussian mixture modeling in the mclust package of R (version 5.4.5; Scrucca et al., 2016). The Bayes information criterion (BIC) was used for model selection and identified a model with five clusters as giving the lowest BIC value.

### Manual analysis of firing properties

All analysis was conducted in Clampfit (Molecular Devices; version 10.7.0.3), with HEKA .dat files first being converted to .txt files in Igorpro (Wavemetrics; version 8.0.4.2) before being imported. Action potentials were detected using a threshold search followed by visual inspection. For parameters describing the action potentials, the first action potential elicited by rheobasic stimulation was used for the measurements. Action potential peaks and thresholds were measured manually by placing cursors on the voltage maximum and the beginning of the rise phase, respectively. The halfwidth was measured as the time between the voltage reaching halfway between the threshold and peak values, and then reaching the same value in the decay phase. The steady state voltage responses of the first four hyperpolarizing steps were used to calculate the input resistance by plotting the voltage against current injected and measuring the gradient. Sag ratio was calculated as the ratio between the trough and steady state of the most hyperpolarizing response. Afterhyperpolarization (AHP) was measured relative to action potential threshold. Amplitude accommodation ratios were calculated by dividing the amplitude of the first peak by that of the last.

### Analysis of tonic currents

To calculate the amplitudes of tonic currents, current traces were first filtered with a 2 kHz eight-pole Bessel filter to visualize synaptic events. Because of the relatively small amplitudes of some of the tonic currents measured in this study, the disappearance of IPSCs was the primary indicator used to locate the onset of the response to the bicuculline. Moving averages of 10-ms windows were then calculated at 100-ms intervals along the current trace to further reduce the noise bandwidth and make changes in the holding current more apparent. These reduced traces were then examined and compared with the original filtered traces to identify outliers occurring because of large synaptic events. Such outliers were subsequently removed from the analysis. Shifts in the holding current were then calculated by taking current averages in 20-s windows at both baseline and in the presence of bicuculline and then taking the difference between these two values. These windows were positioned within 50 s of the onset of the bicuculline block, but their precise locations were adjusted to avoid overlap with any obvious noise or artifacts. Whole-cell capacitance was calculated by calculating the area under the capacitance transient induced by applying a 10-mV voltage step and dividing by the applied voltage.

For root mean square (RMS) calculations, the RMS was calculated for 0.5-s windows by subtracting the mean baseline from that window, then calculating the square of all the sample points and then taking the root of the mean of these values. Differences before and after bicuculline application where again calculated by averaging 20-s windows of these values either side of the time point when the bicuculline block became apparent.

Responses to allopregnanolone, DS2, and MRK-016 were analyzed in a similar manner to the tonic inhibitory currents, with a moving average being applied before the measurement of the induced currents. Current deflections induced by the drugs were visually identified and measured relative to the current baseline before drug application.

### Imaging of labeled neurons

Slices with recorded cells that had been labeled with biocytin during recordings were subsequently fixed and washed in PB (0.1 m, pH 7.2) before being fixed in gelatin (20% in dH_2_O) and recut into ∼60-µm sections. The sections were further washed in 0.3% Triton X-100 (dissolved in tris-buffered saline; TBS) for 3 × 10 min at room temperature before being incubated in streptavidin conjugated to either Alexa Fluor 488 (1:1000) or Cy3 (1:400), overnight at 4°C. They were then washed in Triton X-100 (0.3% in TBS) three times for a total of 60 min before being mounted in Vectashield mounting media (Vector Laboratories). Once mounted they were then either visualized on an epifluorescence microscope (Leica) or taken for imaging on a Zeiss LSM 710 confocal microscope (only those stained with streptavidin conjugated to Alexa Fluor 488). Imaging was conducted using the spectral-scanning mode of the confocal microscope to use linear spectral unmixing (Dickinson et al., 2001) to separate fluorescence of the streptavidin label from that of lipofuscin, which is typically abundant in adult human brain tissue. Images were taken using a plan-apochromat 40×/1.3 oil objective with a zoom of 0.6× to give a resolution of 0.18 µm/pixel and a pinhole size of ∼1 airy unit (30.7 µm, as calculated by the Zen 2008 software), with slices taken at 1-µm intervals. The samples were illuminated with a 488 nm argon laser, and emitted light was collected across the full range of the detector, ranging from 418 to 720 nm across 32 channels using the λ-stack mode within the Zen 2008 software (version 5, Zeiss).

Linear spectral unmixing was conducted on these image stacks using the PoissonNMF plugin for ImageJ (Neher et al., 2009). Spectra for the streptavidin fluor 488 and lipofuscin were generated by drawing regions of interest around structures that could clearly be identified as axon or dendrites and lipofuscin granules respectively. Spectral unmixing was then conducted using these spectra to separate the streptavidin and lipofuscin emissions into two pseudochannels. Denoising of the resulting image was conducted using the non-local means option in DenoiseEM (Roels et al., 2019). In some cases, stitching of multiple frames was also conducted to produce the final result. This was achieved using the pairwise-stitching tool within ImageJ.

Some neurons were reconstructed in two dimensions from one to three ∼60-µm-thick sections after re-cutting the slices, using a 100× oil immersion objective and a drawing tube. In these specimens, biocytin was first visualized by fluorescence then by horseradish peroxidase reaction and osmium tetroxide treatment (Varga et al., 2015).

### Statistics

All statistical tests used are reported in full in the text. These were conducted in OriginPro (2017 version, OriginLab). On graphs, stars correspond to the following *p* values: *p < 0.05, **p < 0.005, ***p < 0.0005. For multiple comparison tests, only *post hoc* tests indicating a significant difference between groups are reported. Datasets were tested for normality and further inspected manually for skew and outliers before selecting the statistical tests indicated.

## Results

### Cell type-specific expression of GABA_A_R subunits, associated with tonic inhibition, in transcriptomic datasets

**Figure 1.**
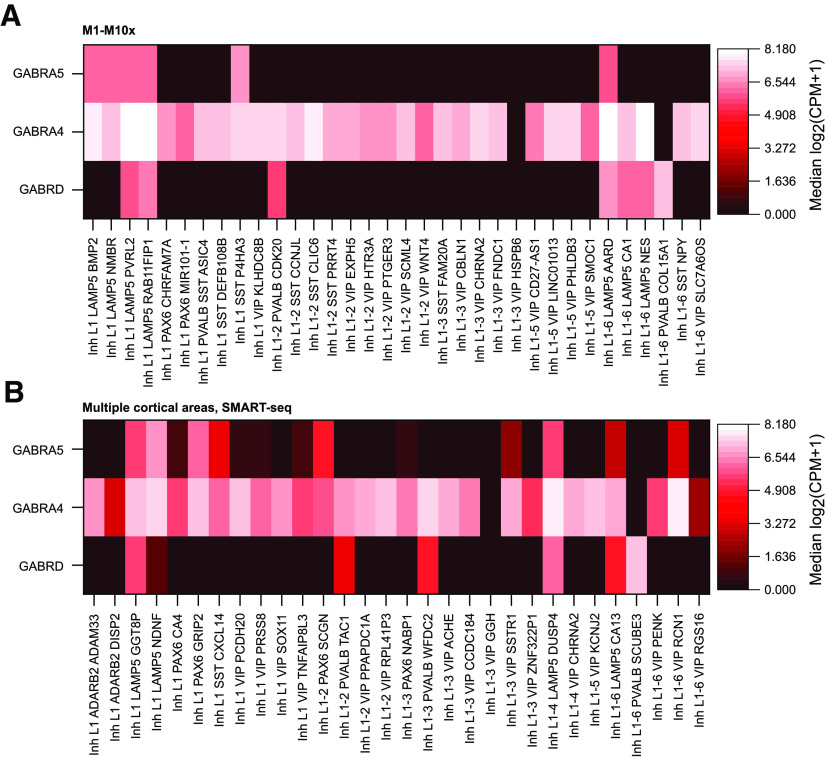
Analysis of the differential expression of GABA_A_R subunits, associated with receptors published previously as mediating tonic inhibition, in the Allen Institute human transcriptomic datasets. Heatmaps of the expression of mRNAs encoding the α4, α5, and δ subunits of the GABA_A_R (*Gabra4*, *Gabra5*, and *Gabrd*, respectively) in two (***A***, ***B***) transcriptomic datasets available at http://celltypes.brain-map.org/rnaseq/. Profiles are only shown for GABAergic interneurons that are indicated as present within layer 1. Data obtained from postmortem and neurosurgical tissue samples. ***A***, Expression profiles of GABA_A_R subunits in tissue taken from human primary motor cortex and processed using the 10× genomics RNA-seq methodology. ***B***, Expression profiles from a dataset covering multiple cortical areas (middle temporal gyrus, anterior cingulate cortex, primary visual cortex, primary motor cortex, primary somatosensory cortex, and primary auditory cortex), profiled using SMART-seq v4. Note that the two datasets differ in their cluster identifiers. CPM - counts per million.

To begin exploring the hypothesis that tonic inhibition may vary among interneuron types in layer 1 of the human neocortex, we first examined transcriptomic profiles produced by the Allen Brain Institute (Hodge et al., 2019; Bakken et al., 2020; BRAIN Initiative Cell Census Network, 2020) to see whether GABA_A_R subunits often present in receptors responsible for tonic inhibition (α4, α5, δ) exhibit any specificity in their expression profiles in layer 1 interneurons (Fig. 1*A*,*B*). Of the three subunits, *GABRA4* showed the broadest expression profile, being found in all but two clusters in two datasets (Fig. 1*A*,*B*). Conversely, *GABRA5* showed a relatively limited expression pattern in the primary motor cortex dataset (Fig. 1*A*), but a less specific profile in a dataset drawn from multiple cortical areas (Fig. 1*B*), suggesting variability in its expression pattern over different cortical areas. However, the profiles of *GABRD* showed similar cell type-specific patterns of expression in both datasets, with the mRNA encoding the δ subunit only being found in subsets of *LAMP5*-positive and *PVALB*-positive neuron clusters (7/34 and 7/30 clusters, respectively; Fig. 1*A*,*B*), with a few of these clusters also showing *GABRA5* co-expression (three and four clusters, respectively; Fig. 1*A*,*B*). It is worth noting, however, that *PVALB-*positive nuclei are relatively rare in layer 1 dissected from human tissue, while *LAMP5 expressing* nuclei are quite common (Hodge et al., 2019). Additionally, three clusters (two *LAMP5*, and one *SST*) in M1, and 10 in the multiple cortical areas dataset also show expression of only *GABRA5*. Interestingly, the *LAMP5* clusters exhibiting both *GABRD* and *GABRA5* expression include the Inh L1–6 *LAMP5 AARD* (Fig. 1*A*) and Inh L1–4 *LAMP5 DUSP4* groups (Fig. 1*B*), which appear to correspond to the transcriptomic profile of the Rosehip cell (Boldog et al., 2018; Hodge et al., 2019), a type of interneuron only identified in human cortex. The other four *LAMP5* clusters exhibiting *GABRD* expression have all been identified as the human homologs of mouse transcriptomic clusters that correspond to neurogliaform cells (BRAIN Initiative Cell Census Network, 2020).

### Clustering of layer 1 cortical interneurons by firing properties

**Figure 2.**
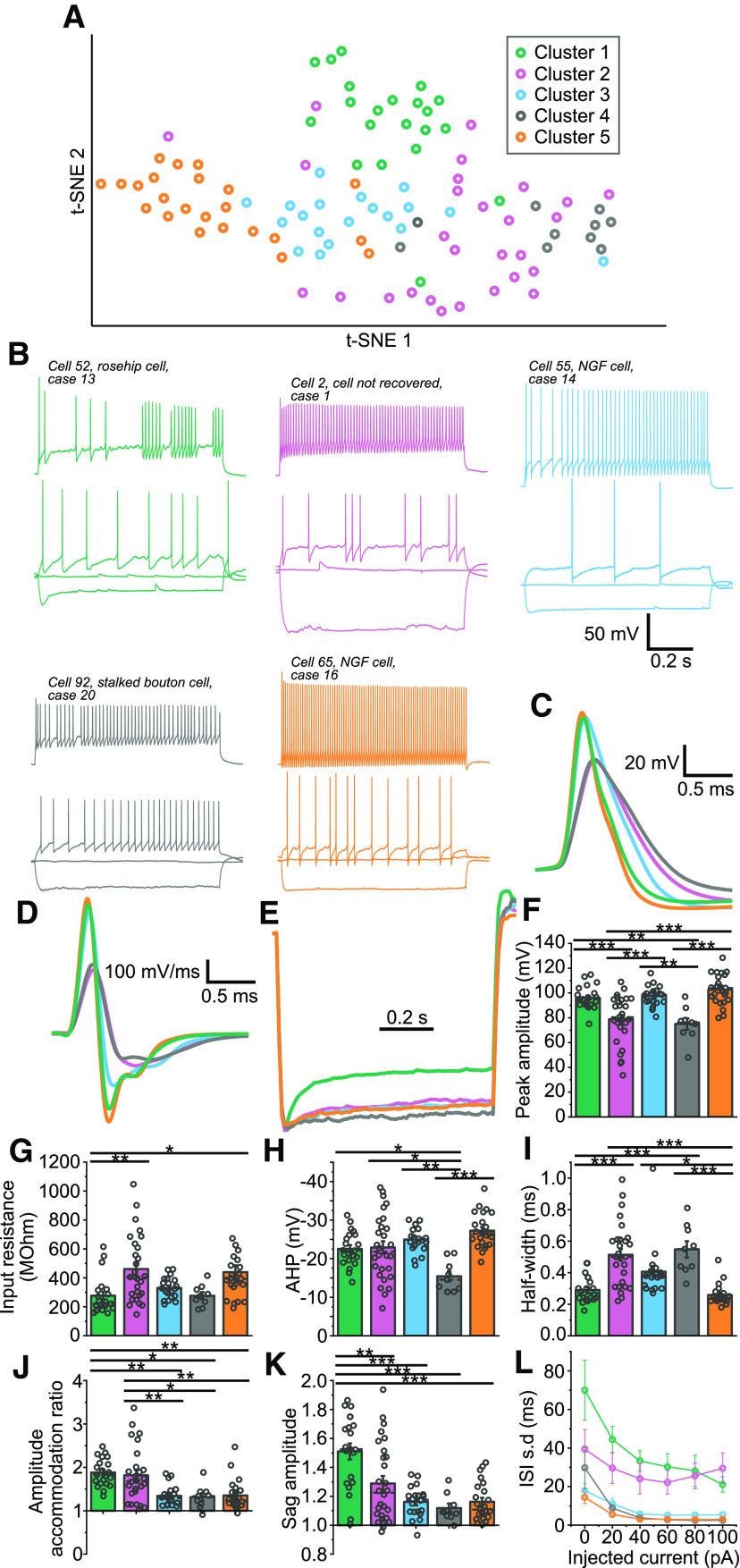
Clustering of human cortical interneurons based on their firing and membrane properties recorded mainly in layer 1. ***A***, t-SNE plot generated from datasets extracted from current-clamp, IV traces using the Allen Brain Institute parameter extraction method. Colors represent the five clusters identified by Gaussian mixture modeling of these datasets (*n* = 101 cells; cluster 1, green; cluster 2, pink; cluster 3, blue; cluster 4, gray; cluster 5, orange). ***B***, Representative example traces of cells from the five clusters. The responses to holding current, holding current minus 100 pA and rheobase current injection are shown as overlaid traces, along with the response to rheobase plus 100 pA (offset for clarity). ***C***, Average waveforms of the first action potential fired in response to stimulation with rheobase current for each of the cells from the five clusters. For each cluster the action potentials of all cells within the cluster were aligned by their thresholds before being averaged to produce the traces shown. ***D***, Average waveforms of the action potentials differentiated with respect to time, from each of the five clusters ***E***, Normalized average voltage responses to current injection with an amplitude of the holding current minus 100 pA. For each cluster individual traces for all the cells included in the cluster were first normalized such that the initial baseline and the peak of the voltage sag take equal values for each cell before averaging the waveforms to produce the displayed traces. ***F–K***, Plots of (***F***) the amplitude of the first action potential peak elicited by stimulation at rheobase (one-way ANOVA: *F*_(96)_ = 14.7, *p* = 2.1 × 10^−9^; Tukey's tests: cluster 1 vs 2, *p* = 0.00032; 2 vs 3, *p* = 0.000086; 1 vs 4, *p* = 0.0020; 3 vs 4, *p* = 0.00077; 2 vs 5, *p* = 1.3 × 10^−7^; 4 vs 5, *p* = 0.000014); (***G***) input resistance (one-way ANOVA: *F*_(96)_ = 5.08, *p* = 0.00094; Tukey's tests: cluster 1 vs 2, *p* = 0.0043; 1 vs 5, *p* = 0.023); (***H***) AHP amplitude of the first action potential (one-way ANOVA: *F*_(96)_ = 7.47, *p* = 0.000028; Tukey's tests: cluster 1 vs 4, *p* = 0.020; 2 vs 4, *p* = 0.0084; 3 vs 4, *p* = 0.00070; 4 vs 5, *p* = 0.0000091); (***I***) half-width of the first action potential (one-way ANOVA: *F*_(96)_ = 15.0, *p* = 1.4 × 10^−9^; Tukey's tests: cluster 1 vs 2, *p* = 6.4 × 10^−6^; 1 vs 4, *p* = 0.00022; 2 vs 5, *p* = 1.0 × 10^−7^; 3 vs 5, *p* = 0.010; 4 vs 5, *p* = 0.000018); (***J***) the amplitude accommodation ratio for rheobase plus 60-pA current injection (calculated as the ratio of the first peak divided by the last; one-way ANOVA: *F*_(94)_ = 9.02, *p* = 0.0000033; Tukey's tests: cluster 1 vs 3, *p* = 0.00075; 2 vs 3, *p* = 0.0023; 1 vs 4, *p* = 0.0086; 2 vs 4, *p* = 0.021; 1 vs 5, *p* = 0.00059; 2 vs 5, *p* = 0.0018); (***K***) the amplitude of the sag of the voltage response elicited by injection of holding current minus 100 pA, represented as the ratio between the peak of the sag and the steady state response (one-way ANOVA: *F*_(96)_ = 11.7, *p* = 8.8 × 10^−8^; Tukey's tests: cluster 1 vs 2, *p* = 0.0026; 1 vs 3, *p* = 3.1 × 10^−6^; 1 vs 4, *p* = 0.000048; 1 vs 5, *p* = 1.5 × 10^−6^). ***L***, The ISI SDs plotted against the amount of current injected relative to rheobase (rheobase = 0 pA). For each cluster, the ISI SDs for each cell in the cluster at a given amount of current injected were averaged. Only traces displaying four or more action potentials were included in these calculations. t-SNE - t-distributed stochastic neighbor embedding; NGF - neurogliaform cell.

To examine whether such transcriptomic heterogeneity translates into differences in the tonic GABA_A_R-mediated currents of layer 1 interneurons, patch-clamp recordings were made from layer 1 cells in slices of human neocortex that had been resected during surgical procedures to treat either brain tumours or temporal lobe epilepsy (Table 1). In order to begin to dissect the diversity of these neurons, firstly the firing properties of the recorded cells were analyzed. This was achieved using the parameter extraction method of Gouwens et al. (2019), a procedure which extracts a large amount of information from the recorded IV response traces using sPCA to analyze the variance in the structures of the voltage responses. Once the scores had been calculated using this method, Gaussian mixture modeling was used to cluster the cells, with BIC minimization identifying a five-cluster model as the most appropriate for this dataset (Fig. 2*A*,*B*).

The five clusters identified by this strategy differed in several features. When comparing the kinetics of the first action potentials fired at rheobase, the clusters appeared to fall into two groups, with clusters 1, 3, and 5 having larger peak amplitudes than clusters 2 and 4 (Fig. 2*C–F*). Clusters 1 and 5 also had smaller mean half-widths (Fig. 2*C*,*D*,*I*). Additionally, action potentials from cells in cluster four tended to have smaller AHPs (Fig. 2*H*) than the other clusters.

In terms of the membrane properties of the cells, there was some variability in the input resistances of the recorded cells, with clusters 2 and 5 displaying significantly higher mean input resistances than cluster 1 (Fig. 2*G*), and a notable number of high resistance cells falling into cluster 2 (Fig. 2*B*,*G*). Additionally, cluster 1 cells tended to display large voltage sags in their responses to hyperpolarizing current injection, a feature also observed in some cluster 2 cells (Fig. 2*B*,*E*,*K*).

Finally, the firing properties of the cells also provide two key distinctions between clusters 1 and 2, and the other cells. Both clusters 1 and 2 displayed irregular firing patterns compared with the regular patterns of the other three clusters (Fig. 2*B*,*L*). Additionally, the SDs of the interspike intervals (ISIs) tended to be higher for cluster 1 than for cluster 2 at rheobase (Fig. 2*L*). The other key difference in the firing was that while most of the recorded cells showed some accommodation in the amplitudes of their action potentials (99/101 cells with ratios greater than one; Fig. 2*J*), this accommodation was on average significantly larger for clusters 1 and 2 (Fig. 2*B*,*J*).

The properties of the clusters identified by this method were subsequently compared with those previously reported for layer 1 interneurons in both human and rodent. Of the groups reported here, cluster 1 displayed the clearest correspondence to any previously reported cell types, displaying both the large hyperpolarizing voltage-sag and irregularity (Fig. 2*B*,*E*,*K*,*L*) of the rosehip cell (Boldog et al., 2018). Conversely, cells with the late-spiking firing-pattern that has previously been associated with neurogliaform cells (Schuman et al., 2019) were found primarily in cluster 3 (8 out of 20 cells), but with some examples in other clusters (2/21 cells in cluster 1, 5/28 in cluster 2, 1/9 in cluster 4, and 2/23 in cluster 5). It is worth noting, however, that some of these properties may have been altered by the use of a high chloride concentration (65 mm), rather than a physiological concentration to facilitate the measurement of the tonic GABA_A_R currents.

### Measurements of the amplitudes of tonic currents of layer 1 cortical interneurons

**Figure 3.**
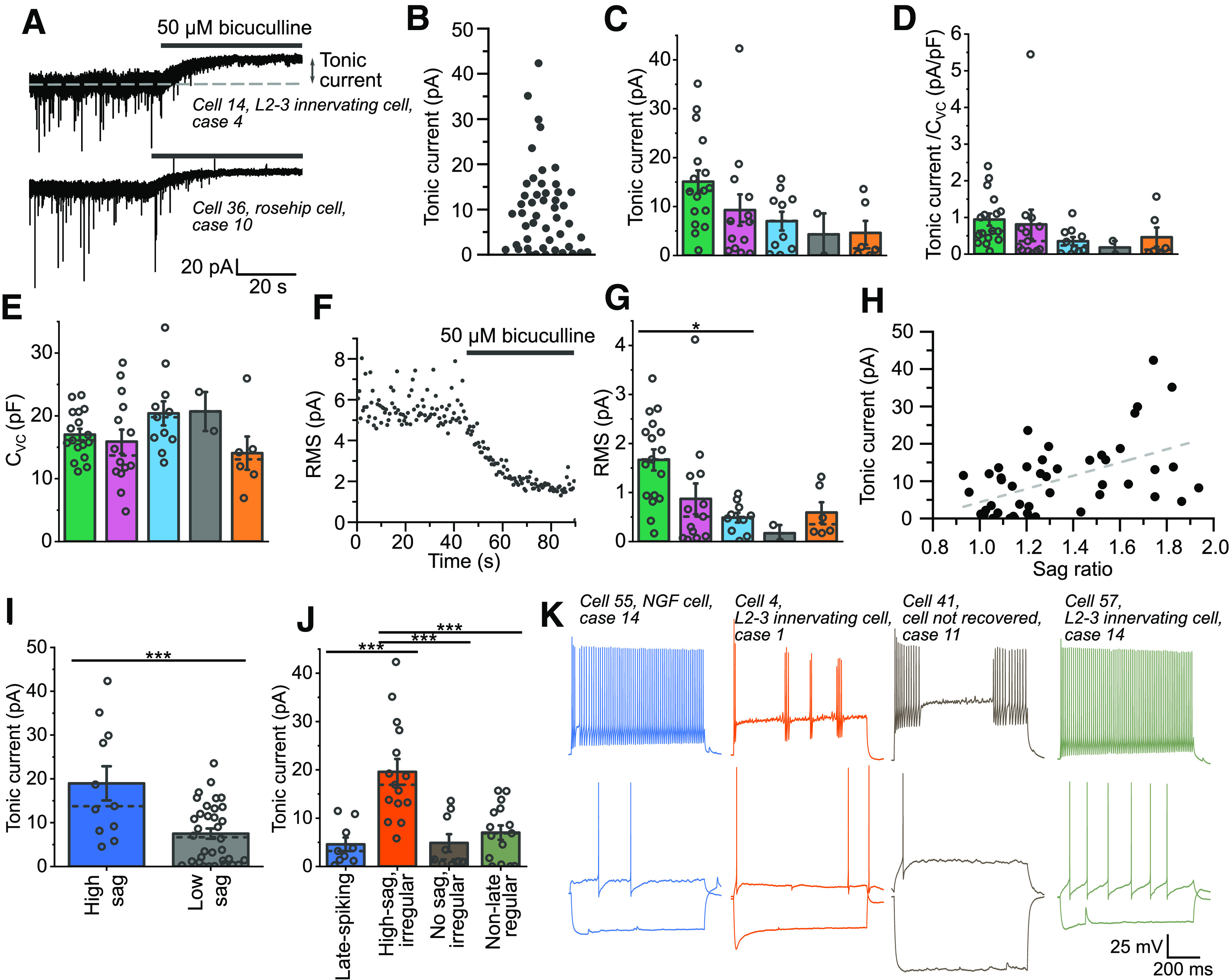
Measurements of tonic GABA_A_R-mediated currents in human cortical interneurons. ***A***, Example current trace demonstrating the response of a cortical interneuron to the application of 50 μm bicuculline methochloride in the presence of bath applied 5 μm GABA. The shift in the holding current (arrows) represents the GABA_A_R-mediated tonic current. The cell was voltage-clamped at −60 mV during the experiment. The downward deflections of current that are blocked by bicuculline application represent spontaneous IPSCs. ***B***, Distribution of the tonic current amplitudes from 48 human cortical interneurons. ***C***, Plot of the mean tonic currents for each of the firing clusters defined in Figure 1 (Kruskal–Wallis ANOVA: χ^2^_(4)_ = 10.9, *p* = 0.028, *n* = 48 cells; Dunn's *post hoc* tests are non-significant for all comparisons; for further multiple regression modeling of these clusters to account for patient variability, see Table 2). ***D***, The ratio of tonic current to whole cell capacitance (C_VC_) measured in the voltage clamp configuration (Kruskal–Wallis ANOVA: χ^2^_(4)_ = 9.29, *p* = 0.054). ***E***, Capacitance measurements made by applying a −10-mV pulse in voltage clamp and calculating the area of the resulting current transient (one-way ANOVA: *F*_(45)_ = 1.62, *p* = 0.18). ***F***, Plot of the root-mean square noise measurements made from the example trace shown in panel ***A***. ***G***, RMS measurements of noise amplitude shifts on the application of bicuculline methochloride for cells grouped according to firing cluster (Kruskal–Wallis ANOVA: χ^2^_(4)_ = 16.3, *p* = 0.0027; Dunn's test: cluster 1 vs 3, *p* = 0.023). ***H***, Plot of the tonic current against the ratio of the sag in the voltage response to a –100-pA current injection from the holding current (see Fig. 2*K*). Dashed line represents a linear-regression fit of this data (slope = 17.7 pA, *t* = 4.1, *p* = 0.00014, Pearson's *r* = 0.52). ***I***, Comparison of tonic currents recorded from cells with sag ratios >1.6 against all other cells (*t*_(43)_ = 3.84, *p* = 0.00039). ***J***, Comparison of tonic currents across cells grouped using a manual Petilla-convention cell firing classification (one-way ANOVA: *F*_(44)_ = 12.6, *p* = 0.0000046; Tukey's tests: late spiking vs high-sag/irregular, *p* = 0.000086; no-sag/irregular vs high-sag/irregular, *p* = 0.00015; non-late/regular vs high-sag/irregular, *p* = 0.00014). ***K***, Example traces from each of the groupings in panel ***J***, displaying the voltage responses to stimulation at rheobase, rheobase minus 100 pA, and rheobase plus 100 pA.

To begin examining whether different types of layer 1 interneurons vary in their inhibitory tonic conductances, measurements were made of the shifts in the holding currents on application of bicuculline (50 μm) to voltage-clamped cells in the presence of bath applied GABA (5 μm) and the ionotropic glutamate receptor blockers NBQX (20 μm) and AP-V (50 μm; Fig. 3*A*). All recordings were made from submersed slices. The tonic currents recorded here are largely in accordance with those previously reported for human cortical interneurons (Scimemi et al., 2006), with a mean of 10.07 ± 1.39 pA (*n* = 48; Fig. 3*B*). However, quite large variability was observed in these values, with a range of 0.01–42.3 pA (Fig. 3*B*). When grouped according to the clustering of cells by their firing properties, cluster 1 showed the highest mean tonic currents, with a value of 15.1 ± 2.3 pA (*n* = 17 cells; Fig. 3*C*). A similar trend was observed when the tonic currents were normalized by whole cell capacitance to account for potential differences in the surface area of the voltage-clamped cell membrane (Golowasch et al., 2009; Taylor, 2012; Fig. 3*D*,*E*). To further confirm this distribution, the change in the root mean square (RMS) of the noise on bicuculline applications was also calculated for each of the cells (Fig. 3*F*,*G*). Such changes in the amplitude of the noise in the recording result from a reduction in channels fluctuating between their open and closed states and provide an alternative measure of the amplitude of tonic inhibition (Glykys and Mody, 2007; Bright and Smart, 2013). These changes in the noise amplitude also showed a similar pattern between clusters, with cluster 1 tending to be highest (1.7 pA). It is also worth noting that the large outlier in cluster 2 also displays properties similar to cluster 1 cells, having a large sag ratio and irregular firing (firing shown in Fig. 3*K*, orange trace). Cluster 1 is primarily distinguished from the others by the presence of a large voltage sag, reminiscent of the recently described rosehip cell in layer one of human cortex (Boldog et al., 2018). The measured tonic currents were therefore plotted against the sag ratio, revealing that cells with larger sag ratios do indeed tend to exhibit larger tonic conductances (Fig. 3*H*). When applying a criterion for cells responding with a high voltage sag similar to that described as a criterion for the identification of rosehip cells (Boldog et al., 2018), a significant difference between cells showing a high or low sag was found (Fig. 3*I*).

**Table 2.** Comparison of multiple regression model parameters for models using the classification of interneurons by firing cluster

	Model 1	Model 2	Model 3	Model 4	Model 5	Model 6
Intercept	−5.3 ± 8.5, *p* = *0.53*	−13.9 ± 8.9, *p* = *0.13*	−12.0 ± 8.7, *p* = *0.18*	−14.0 ± 9.1, *p* = *0.13*	−16.2 ± 10.4, *p* = *0.13*	1.9 ± 13.6, *p* = *0.89*
Age	0.25 ± 0.16, *p* = *0.14*	0.33 ± 0.16, *p* = 0.044*	0.33 ± 0.16, *p* = 0.044*	0.35 ± 0.16, *p* = 0.036*	0.34 ± 0.18, *p* = *0.064*	0.27 ± 0.18, *p* = *0.15*
Sex (male = 1)	4.0 ± 2.8, *p* = *0.16*	2.1 ± 2.8, *p* = *0.46*	2.1 ± 2.8, *p* = *0.46*	1.8 ± 3.0, *p* = *0.56*	2.1 ± 3.3, *p* = *0.54*	1.9 ± 3.7, *p* = *0.61*
Cluster 1	--	10.6 ± 4.2, *p* = 0.016*	8.7 ± 3.5, *p* = 0.018*	10.3 ± 4.3, *p* = 0.022*	11.5 ± 5.0, *p* = 0.027*	10.9 ± 4.5, *p* = 0.020*
Cluster 2	--	5.2 ± 4.3, *p* = *0.24*	3.3 ± 3.7, *p* = *0.38*	4.1 ± 4.6, *p* = *0.37*	6.5 ± 5.4, *p* = *0.23*	6.3 ± 4.8, *p* = *0.20*
Cluster 3	--	1.9 ± 4.5, *p* = *0.68*	--	1.5 ± 4.6, *p* = *0.75*	2.7 ± 5.0, *p* = *0.59*	4.3 ± 5.2, *p* = *0.42*
Cluster 4	--	−0.14 ± 7.3, *p* = *0.98*	−2.0 ± 7.0, *p* = *0.78*	−1.6 ± 7.8, *p* = *0.84*	0.89 ± 8.1, *p* = *0.91*	6.6 ± 9.4, *p* = *0.49*
Cluster 5	--	--	−1.9 ± 4.5, *p* = *0.68*	--	--	--
Temporal lobe epilepsy	--	--	--	2.1 ± 5.1, *p* = *0.69*	--	--
Glioblastoma	--	--	--	−1.5 ± 3.2, *p* = *0.66*	--	--
Cortical infiltration (MRI)	--	--	--	--	1.6 ± 3.9, *p* = *0.69*	--
Hemisphere (left = 1)	--	--	--	--	0.64 ± 3.9, *p* = *0.87*	--
Frontal	--	--	--	--	−0.0027 ± 3.1, *p* = *0.99*	--
Parietal	--	--	--	--	3.1 ± 6.0, *p* = *0.61*	--
Seizures in last year	--	--	--	--	--	−7.2 ± 6.1, *p* = *0.25*
Seizures more than a year ago	--	--	--	--	--	−1.5 ± 4.7, *p* = *0.75*
Dexamethasone presurgery	--	--	--	--	--	−6.6 ± 4.4, *p* = *0.14*
Dexamethasone at time of surgery	--	--	--	--	--	−8.0 ± 5.6, *p* = *0.16*
Levetiracetam	--	--	--	--	--	−4.2 ± 4.9, *p* = *0.40*
df	45	41	41	39	37	36
Residual sum of squares	3894	3131	3131	3079	3074	2890
*R*^2^ (COD)	0.11	0.28	0.28	0.29	0.30	0.34
Adj. *R*^2^	0.068	0.18	0.18	0.15	0.11	0.14
ANOVA						
F	2.71	2.7	2.7	2.0	1.6	1.7
p	0.077	0.027*	0.027*	0.068	0.16	0.12

The dependent variable is tonic current. All independent variables are given in the first column. Coefficients for each model are given in pA along with SEs and with the associated *p* values given below in italics. Summary statistics for each model are given at the bottom of the table. For sex and hemisphere, coefficients were arbitrarily calculated such that they represent the predicted change in tonic amplitude if the patient was male or the tissue was taken from the left hemisphere, respectively.

A similar result was seen when the cells were manually divided by a more classical Petilla-style classification (Petilla Interneuron Nomenclature Group et al., 2008; Fig. 3*J*,*K*). This was achieved by first dividing the cells into regular and irregular firing, based on visual inspection of their firing patterns. The regular firing cells were further divided based on whether they showed a delayed firing at rheobasic levels of stimulation. While an examination of the irregular-firing cells appeared to show two quite distinct groups, one with the high amplitude sag typical of rosehip cells in layer 1 (Boldog et al., 2018), and another consisting of cells with high input resistances and generally lacking voltage sags, as well as a tendency toward bursting modes of firing (Fig. 3*K*). In this classification, rosehip-like group of cells displaying large sags and irregular firing patterns displayed significantly larger tonic currents than all other groups (Fig. 3*J*).

### Comparison of IV responses recorded before and during the application of receptor antagonists

**Figure 4.**
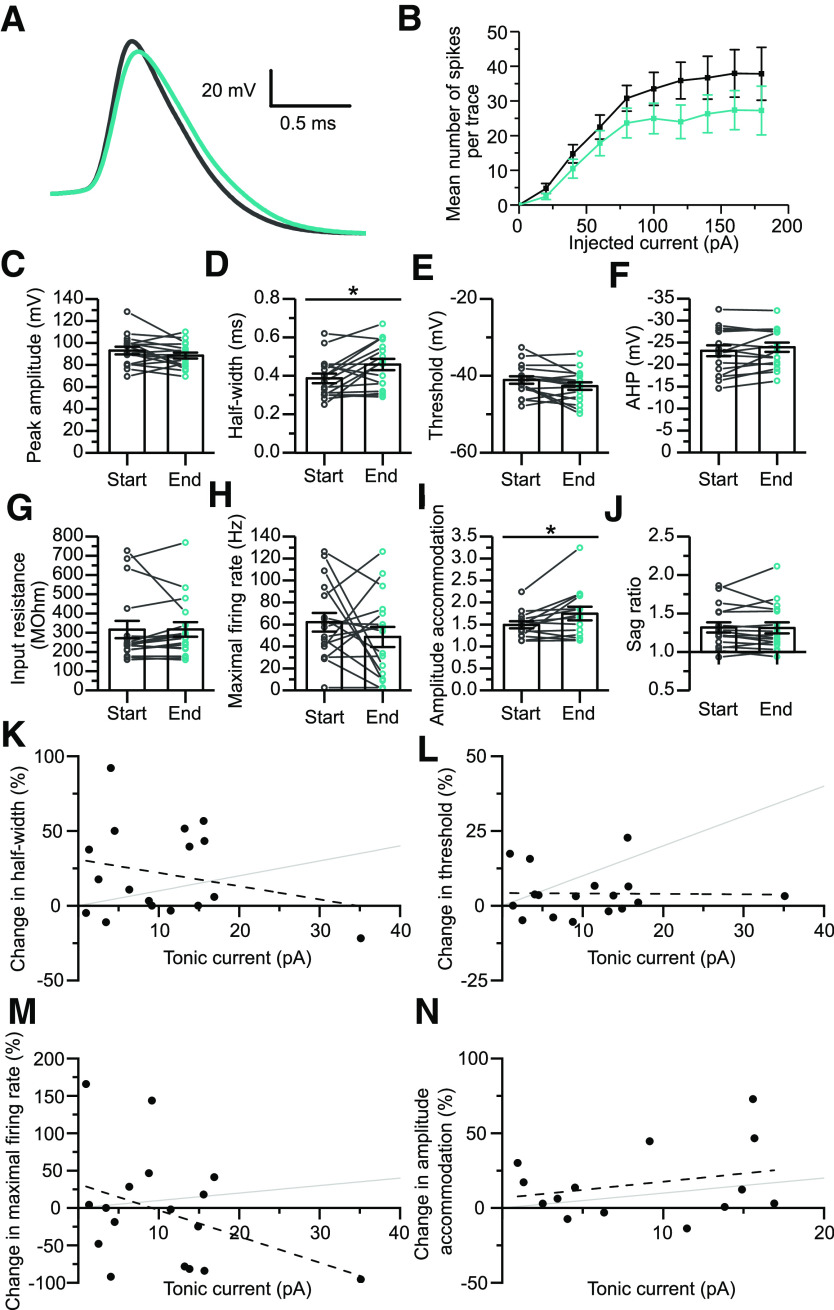
Comparison of membrane and firing properties of interneurons in the presence or absence of the GABA_A_ and ionotropic glutamate receptor antagonists. ***A***, Averages of the first action potential waveforms for 17 of the cells for which tonic inhibitory currents were measured. Responses from the start of the experiment (black), and the end of the experiment (blue; measured in the presence of 50 μm bicuculline, 20 μm NBQX, and 50 μm APV) are shown. ***B***, Relationship of the average firing frequency to depolarizing current injection (20-pA steps), before (black), and after (blue) the application of receptor antagonists. The injected current was normalized such that rheobase is equal to 20 pA on the *x*-axis. ***C–F***, Plots of the peak amplitude (*t* test: *t*_(16)_ = 1.589, *p* = 0.132), half-width (*t* test: *t*_(16)_ = −2.913, *p* = 0.0102), threshold (*t* test: *t*_(16)_ = 2.106, *p* = 0.0513), and AHP amplitude (*t* test: *t*_(16)_ = 1.569, *p* = 0.136) of the first action potential recorded in response to rheobasic stimulation of each cell, measured before (start) and during (end) the application of receptor antagonists. ***G–J***, Measurements of the input resistance (*t* test: *t*_(16)_ = −0.0179, *p* = 0.986), maximal firing rate (*t* test: *t*_(16)_ = 1.168, *p* = 0.260), amplitude accommodation (*t* test: *t*_(13)_ = −2.55, *p* = 0.0240), and sag ratio (*t* test: *t*_(16)_ = 0.164, *p* = 0.871) calculated for each cell at the start of the experiment and in the presence of receptor antagonists. ***K–N***, Plots of the change in half-width (Pearson's *r* = –0.25; intercept = 30.9 ± 12.0%, *t* = 2.6, *p* = 0.022; slope = –0.89 ± 0.91%/pA, *t* = –0.98, *p* = 0.34), threshold (Pearson's *r* = –0.015; intercept = 4.3 ± 3.2%, *t* = 1.32, *p* = 0.21; slope = –0.015 ± 0.24%/pA, *t* = –0.060, *p* = 0.95), maximal firing rate (Pearson's *r* = −0.39; intercept = 32.1 ± 28.8%, *t* = 1.1, *p* = 0.28; slope = −3.5 ± 2.2%/pA, *t* = −1.6, *p* = 0.13), and amplitude accommodation (Pearson's *r* = 0.27; intercept = 6.63 ± 11.7%, *t* = 0.56, *p* = 0.58; slope = 1.1 ± 1.1%/pA, *t* = 0.97, *p* = 0.35) between the start of the experiment and in the presence of receptor antagonists, against the amplitude of the tonic current. Dashed lines represent a linear fit of the data, gray lines represent a gradient of 1%/pA for comparison.

Given that tonic currents could potentially alter the firing properties of the cells they were recorded from, it is possible that some of the segregation of these cells into clusters based on their firing properties could have been influenced by differences in their tonic conductances. To investigate this, IV responses were again recorded while the receptor antagonists and GABA (50 μm AP-V, 20 μm NBQX, 50 μm bicuculline, and 5 μm GABA) were still present. It is important to note here that these recordings were made using high-chloride internal solutions, so the comparisons reported here are unlikely to reflect the physiological effects of tonic inhibition on the firing properties of the cells. The shapes of the action potentials recorded under these conditions were largely unchanged, other than a slight increase in their half-widths (Fig. 4*A*,*D*). No significant alterations in peak amplitude, threshold, or AHP amplitude were observed (Fig. 4*A*,*C*,*E*,*F*). Perhaps unexpectedly, the input resistance of the cells also remained unchanged by these receptor antagonists (Fig. 4*G*), this was likely because the exogenous GABA used to induce the tonic currents was not applied until after the measurement of the baseline IV response and would therefore suggest that the residual GABA that was present in the slices only induced small conductances in those cells tested.

While the maximum firing rate of the cells seemed to often change after the application of the antagonists, these changes were highly variable and bidirectional so no significant alteration in the mean was observed (Fig. 4*B*,*H*). Conversely, a slight but significant increase in the amplitude accommodation was observed (Fig. 4*I*). Importantly, the sag ratio, a key parameter distinguishing cluster 1 cells from all other clusters, showed no significant alteration in the presence of the channel blockers (Fig. 4*J*). Overall, it seems unlikely that the segregation of the cells by firing was influenced by differences in the tonic currents experienced by the cells, given that key parameters appear unchanged and that the only significant differences are relatively small when compared with those observed between clusters. Furthermore, of the parameters that exhibited some change, the magnitude of those changes did not correlate with the amplitudes of the tonic currents recorded from the same cell (Fig. 4*K–N*). These comparisons would therefore support the use of firing parameters, and in particular the sag ratios, to segregate the cells.

### Anatomical analysis of interneurons and correlations with firing and tonic currents

To further characterize differences in tonic GABA_A_R currents, recovered cells were classified by their axonal patterns. Of the 101 cells recorded in and close to layer 1, 67 interneurons were recovered with sufficient axon for comparison, which was the most prominent distinguishing feature of the labeled neurons. An additional 17 neurons were partially recovered for microscopic analysis including eight cells with soma and dendrites only and nine neurons with dendrites and/or soma, but too little axon for categorization, as well as an additional five axons from slices in which the somata and dendrites were lost during processing. As determined from the DIC images and biocytin labeling, the cell bodies of neurons were either in layer 1 (*n* = 95), or in layer 2 (*n* = 5) at the border with layer 1.

**Figure 5.**
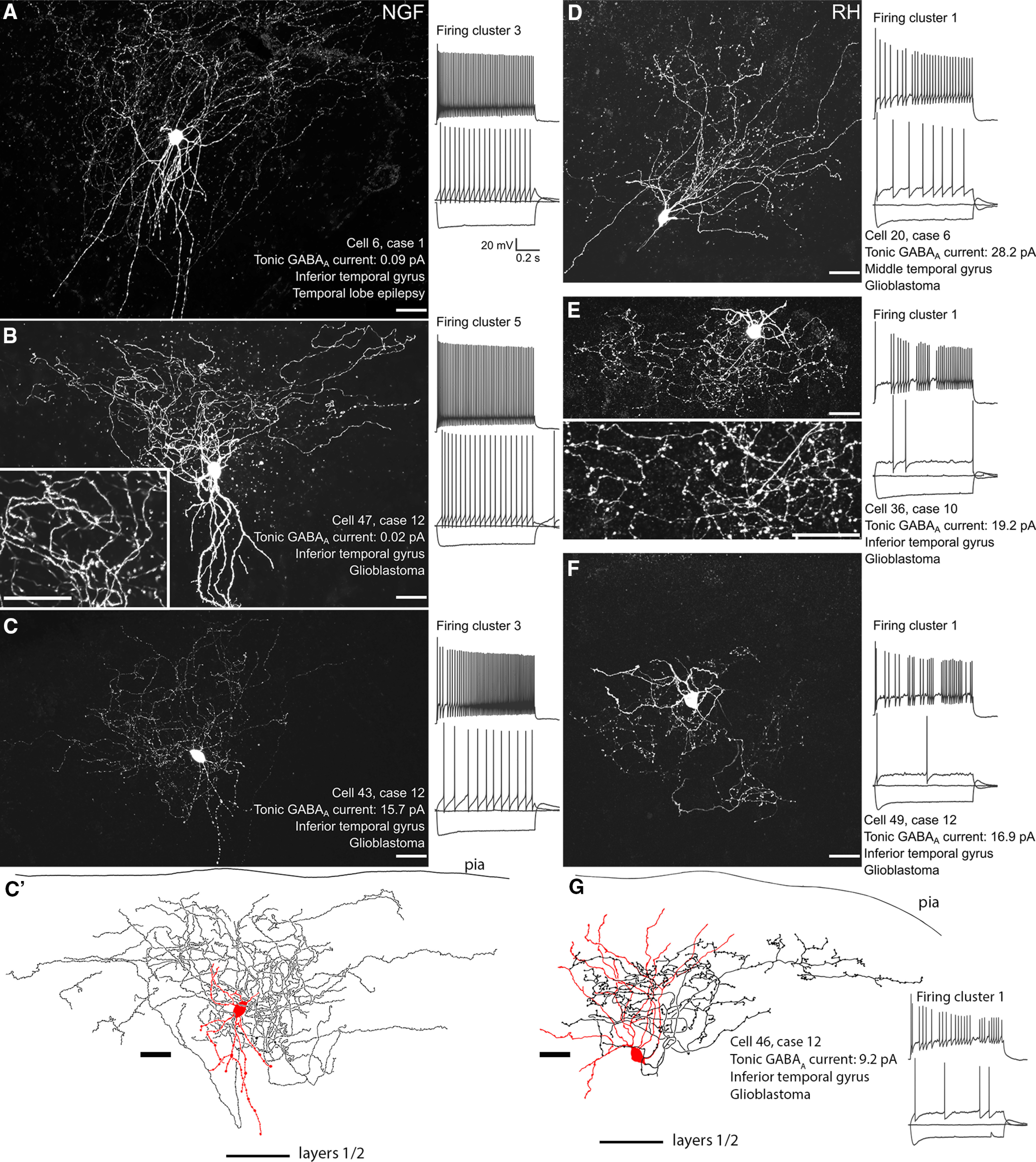
Examples of neurons visually identified as either neurogliaform (NGF) or rosehip (RH) cells in layer 1. ***A–C***, Maximal intensity projections of confocal z-stack images of labeled neurogliaform cells as shown by their axonal distribution. Cells were labeled with biocytin in the patch pipette and visualized by fluorophore conjugated streptavidin in single sections. Spectral imaging was used to separate the fluorescent signal of streptavidin from lipofuscin autofluorescence (see methods). ***C'***, Two-dimensional drawing tube image of the cell shown in *c* reconstructed from three sections; soma and dendrites in red, the axon in black; position shown between the pia and the layers 1 and 2 boundary. ***D–G***, Images of labeled cells identified as rosehip cells; the reconstructed cell in ***G*** is shown from two sections. Insets in ***B***, ***E*** show characteristic differences in axonal shape and bouton size between the two cell types. To the right of each image, the response of the cell is shown to holding current, holding current minus 100 pA and rheobase current injection as overlaid traces, along with the response to rheobase plus 60 pA (offset for clarity). Note the differences in spike patterns and amplitude. Scale bars: 25 µm (images); voltage traces 20 mV and 200 ms (scale bars in ***A*** apply to all).

**Figure 6.**
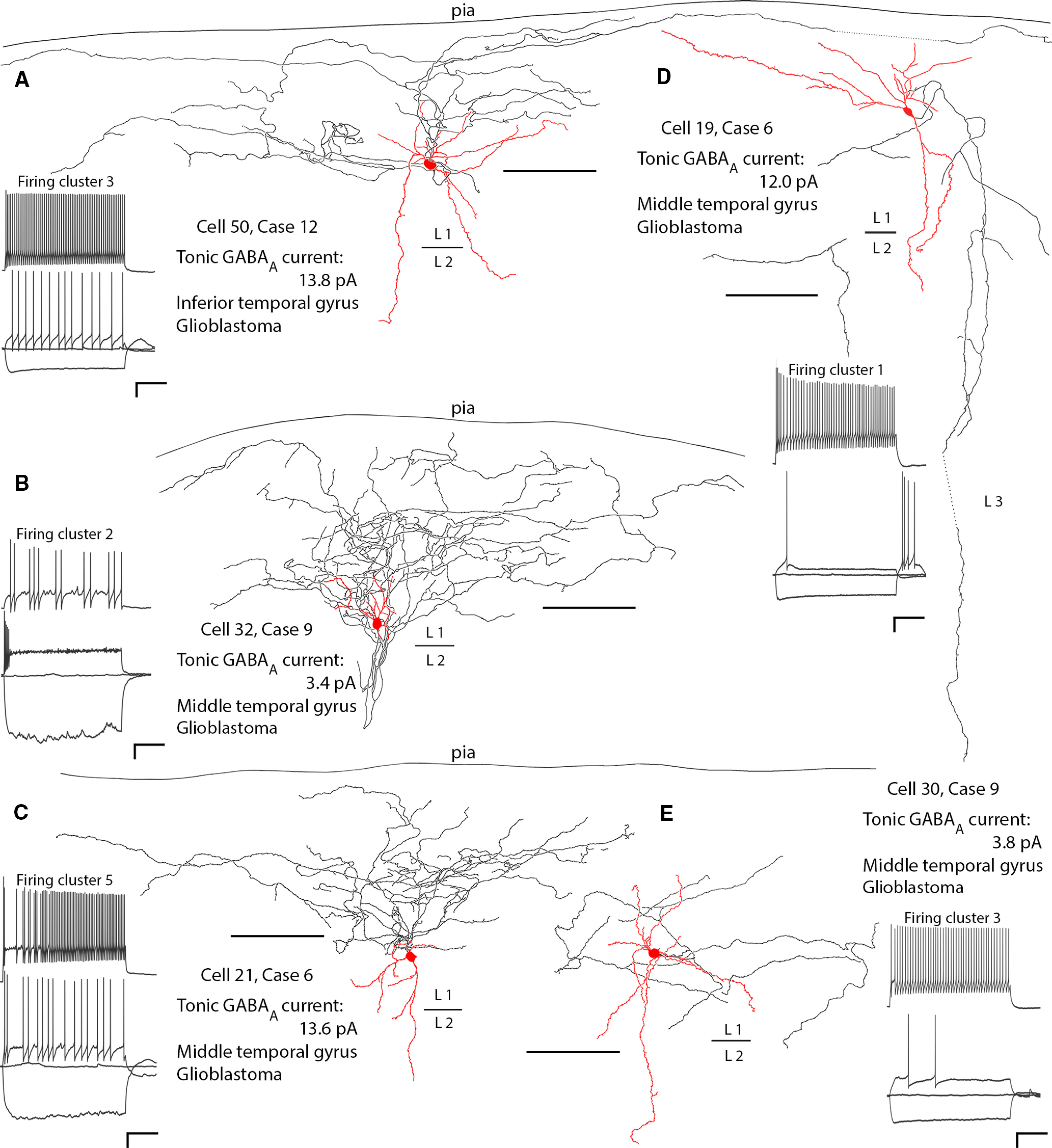
Reconstructions of the dendritic (red) and axonal (black) trees of diverse interneurons in layer 1 with distinct firing patterns and differing tonic GABA_A_R-mediated currents. ***A***, Neurogliaform cell with wide subpial wavy axon collaterals; the axon restricted to layer 1. ***B***, Neuron with mostly ascending dendrites and recurrent dense axon with medium size boutons, many of them on stalks (data not shown); the axon restricted to layer 1. ***C***, Neuron with mostly descending dendrites and dense ascending axon with medium sized boutons, many of them on stalks (data not shown); axon restricted to layer 1. ***D***, Bitufted neuron with spiny dendrites and descending axon to layers 2 and 3. ***E***, Multipolar dendritic neuron with sparse axon mostly in layer 1. Each cell is shown from one to three ∼60-µm-thick sections; many processes are truncated (not indicated). Scale bars: 100 µm (images); voltage traces 20 mV and 200 ms (scale bars set uniformly).

Of these recovered cells, we have identified 13 rosehip cells based on the high density of large boutons along frequently branching axons within a restricted volume around the cell body (Fig. 5*D–G*). We have identified 22 neurogliaform cells with cell bodies in layer 1 (Fig. 5*A–C*) and an additional one with cell body in layer 2. These neurons are GABAergic (Kisvárday et al., 1990) and differed from rosehip cells, by having much smaller boutons with lower frequency along very densely branching axonal plexus, with individual branches showing a “wavy” appearance. The axons of the recorded neurogliaform cells were mostly located in layer 1, rarely with small contributions to layer 2. Within layer one, some neurogliaform cells exclusively innervated the subpial one third or one half (Figs. 5*A*,*B*, 6*A*), or the middle of layer 1, but some cells did not show sublayer selectivity and their axons occupied most of layer 1 (Fig. 5*C*).

These classifications were in broad agreement with the classification of cells based on their firing, with 11/13 of the rosehip cells clustering into cluster 1, a cluster of cells showing the high voltage sags and irregularity typical of rosehip cells in layer 1 (Boldog et al., 2018), along with one in cluster 2, and one in cluster 3. Conversely, the majority of the neurogliaform cells were split between clusters 3 (eight cells) and 5 (11 cells), with the remainder spread between cluster 2 (two cells), and cluster 4 (two cells).

In addition to the rosehip and neurogliaform cells, 31 interneurons had highly variable axons different from these two cell types (Fig. 6*B–E*). Neurons in the most numerous group (*n* = 13) resembled neurogliaform cells in having high density axons and boutons in layer 1, but their boutons were larger than those of neurogliaform cells and frequently on short 0.2- to 1-μm-long stalks, hence we refer to them as “stalked-bouton cells” (Fig. 6*B*,*C*). Some of their axons ran laterally over hundreds of micrometers and could be only in the subpial one third of layer 1. An additional 12 neurons significantly or mainly innervated layers 2–3 in addition to layer 1, including four basket cells with boutons around the cell bodies of other neurons in layer 2, one Martinotti cell with cell body in layer 2 and seven cells with descending axons (Fig. 6*D*). Five neurons had straight rarely branching axons with relatively low density of boutons in layer 1 (Fig. 6*E*) and one cell had a unique subpial axon. The dendritic fields of some of these latter groups of cells were larger than those of rosehip and neurogliaform cells, and several of them had densely spiny dendrites. Of these other cells, interestingly the firing of several (5/12) of the layer 2–3 innervating cells was qualitatively similar to that of the rosehip cells, displaying large sags and irregularity (Fig. 3*K*, orange trace). Consistent with this, the majority of these cells (9/12) were found in clusters 1, and 2, showing either rosehip-like firing patterns, or in two cases displaying relatively regular firing but with some voltage sag and clear depolarizing humps at rheobase in addition to strong rebound firing after hyperpolarizing current injection (Fig. 6*D*). Conversely, the cells identified with stalked boutons tended to fall in clusters 3 and 5 (9/13 cells), but often displayed high input resistances and some irregularity in their firing (Fig. 6*B*,*C*).

**Figure 7.**
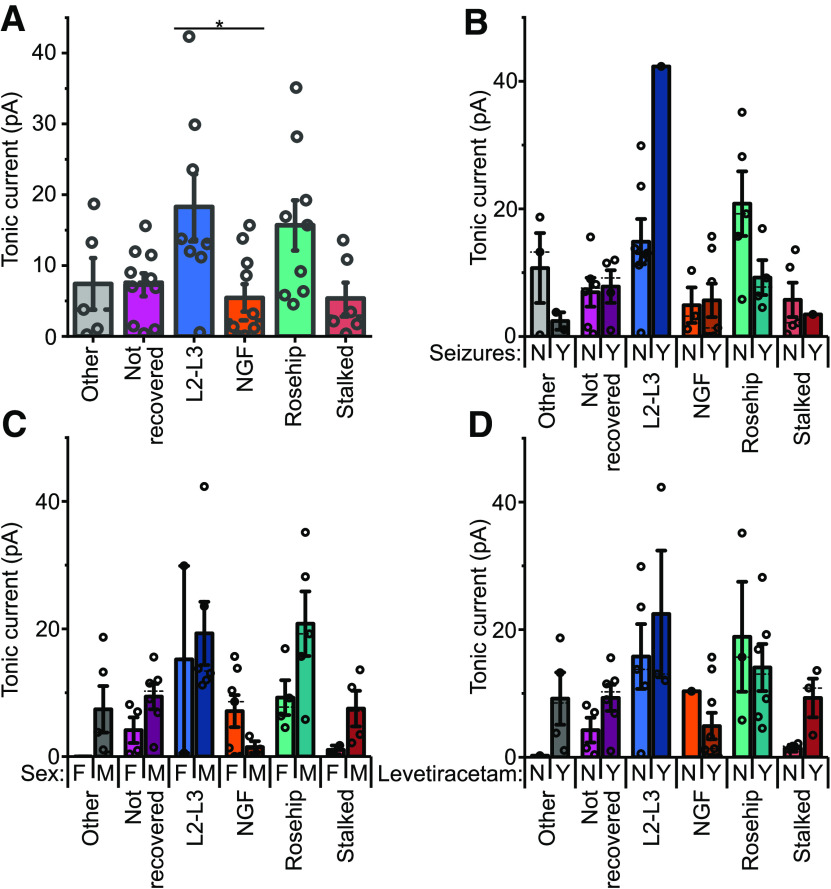
Comparison of tonic inhibitory currents in cell types defined by their axons. ***A***, Tonic current amplitudes plotted with cells grouped according to axonal classification (as described in the Results; see Figs. 6, 7). L2–L3, cells with axons innervating layers 2 and 3; NGF, neurogliaform cells; “stalked,” cells displaying frequent stalked axonal boutons (one-way ANOVA: *F*_(42)_ = 3.50, *p* = 0.0098; Tukey's test: L2–3 innervating cells vs neurogliaform, *p* = 0.032; for further multiple regression modeling of these data to account for patient variability, see Table 3). ***B***, Tonic currents plotted against axonal classification divided by whether or not the patient had a history of seizures in the year preceding the surgery (two-way ANOVA: anatomy, *F*_(5)_ = 6.5, *p* = 0.00021; seizures, *F*_(1)_ = 0.19, *p* = 0.67; interaction, *F*_(5)_ = 3.68, *p* = 0.0086). ***C***, Tonic currents plotted against axonal classification divided by the sex of the patient (two-way ANOVA: anatomy, *F*_(5)_ = 4.35, *p* = 0.00336; sex, *F*_(1)_ = 4.69, *p* = 0.037, interaction, *F*_(5)_ = 1.99, *p* = 0.10). ***D***, Tonic currents plotted against axonal classification divided by whether the patient had been treated with levetiracetam (two-way ANOVA: anatomy, *F*_(5)_ = 3.36, *p* = 0.014; levetiracetam, *F*_(1)_ = 0.99, *p* = 0.33; interaction, *F*_(5)_ = 0.76, *p* = 0.58). For ***B–D***: Y, yes; N, no; M, male; F, female.

When comparing the tonic currents of cells classified by their axons, the layer 2–3 innervating cells displayed significantly higher tonic currents than neurogliaform cells, and also had a trend of having higher means than all groupings other than the rosehip cells, which also displayed a non-significant trend toward having higher mean tonic currents than the other groups (Fig. 7*A*). However, they also displayed more variance than the other groups (Fig. 7*A*). In the case of the layer 2–3 innervating cells, this variation is perhaps explained by the variety of cells included in this group, as shown by their differing axons. Conversely, rosehip cells represent a relatively well-defined group of cells similar in their axons and firing. Additionally, it is important to note that the cells recorded here originate from patients with varying pathology, and previous drug treatments. The tonic currents of these various cell types were therefore further broken down by patient parameters to investigate possible sources of this variation (Fig. 7*B–D*). Interestingly, within the rosehip group, the tonic currents recorded in tissue taken from patients with seizures did appear to show a non-significant trend toward a lower mean value than those from patients without seizures (Fig. 7*B*). However, further examination also showed that of the seizure patients from which rosehip cells with tonic currents were recorded, all happened to be female while all patients without a history of seizures that rosehip cells were recorded from were male (Fig. 7*C*), suggesting that a sex difference could also be a plausible explanation for some of this variability in the tonic currents of rosehip cells. Conversely, levetiracetam usage, which was both given to patients to treat seizures and prophylactically to some cancer patients, did not appear to explain the variation (Fig. 7*D*).

**Table 3.** Comparison of multiple regression model parameters for models using the classification of interneurons by axonal morphology

	Model 7	Model 8	Model 9	Model 10	Model 11	Model 12	Model 13
Intercept	7.3 ± 2.2, *p* = 0.0017*	−12.5 ± 7.5, *p* = *0.11*	−13.6 ± 7.8, *p* = *0.087*	−13.7 ± 7.7, *p* = *0.085*	- 11.4 ± 7.7, *p* = *0.15*	0.68 ± 11.1, *p* = *0.95*	17.9 ± 19.4, *p* = *0.36*
Age	--	0.34 ± 0.14, *p* = 0.022*	0.35 ± 0.14, *p* = 0.020*	0.37 ± 0.15, *p* = 0.016*	0.29 ± 0.15, *p* = *0.065*	0.43 ± 0.16, *p* = 0.011*	0.46 ± 0.17, *p* = 0.013*
Sex (male = 1)	--	2.8 ± 2.5, *p* = *0.27*	3.2 ± 2.6, *p* = *0.23*	2.7 ± 2.9, *p* = *0.36*	4.6 ± 2.8, *p* = *0.12*	−1.2 ± 3.3, *p* = *0.72*	3.1 ± 4.2, *p* = *0.47*
Rosehip	8.3 ± 3.6, *p* = 0.024*	8.7 ± 3.4, *p* = 0.014*	9.1 ± 3.7, *p* = 0.019*	9.8 ± 3.6, *p* = 0.009*	8.3 ± 3.6, *p* = 0.025*	9.3 ± 3.8, *p* = 0.018*	8.8 ± 4.1, *p* = 0.039*
Neurogliaform	−1.9 ± 3.5. *p* = *0.59*	−0.52 ± 3.4, *p* = *0.88*	--	0.039 ± 3.5, *p* = *0.99*	−0.15 ± 3.7, *p* = *0.97*	−0.63 ± 3.6, *p* = *0.86*	0.88 ± 4.0, *p* = *0.83*
Layer 2/3 innervating	11.0 ± 3.7, *p* = 0.0050*	11.6 ± 3.5, *p* = 0.0017**	12.0 ± 4.0, *p* = 0.0043**	12.1 ± 3.5, *p* = 0.0015**	14.4 ± 3.9, *p* = 0.00076**	14.2 ± 3.7, *p* = 0.00046***	16.7 ± 4.2, *p* = 0.00038***
Stalked boutons	−2.0 ± 4.1, *p* = *0.63*	−3.2 ± 3.9, *p* = *0.41*	−2.9 ± 4.2, *p* = *0.50*	−2.1 ± 4.1, *p* = *0.61*	−0.45 ± 4.4, *p* = *0.92*	−1.5 ± 4.0, *p* = *0.70*	0.48 ± 4.5, *p* = *0.92*
Other	--	--	−1.6 ± 4.7, *p* = *0.74*	--	--	--	--
Not recovered	--	--	1.3 ± 3.7, *p* = *0.72*	--	--	--	--
Temporal lobe epilepsy	--	--	--	2.7 ± 4.5, *p* = *0.56*	--	--	0.39 ± 12.7, *p* = *0.98*
Glioblastoma	--	--	--	−1.7 ± 2.9, *p* = *0.56*	--	--	−9.2 ± 4.2, *p* = 0.038*
Cortical infiltration (MRI)	--	--	--	--	4.2 ± 3.3, *p* = *0.21*	--	1.9 ± 6.8, *p* = *0.78*
Hemisphere (left = 1)	--	--	--	--	0.045 ± 3.2, *p* = *0.99*	--	2.6 ± 4.4, *p* = *0.56*
Frontal	--	--	--	--	−2.7 ± 2.8, *p* = *0.35*	--	0.64 ± 5.3, *p* = *0.90*
Parietal	--	--	--	--	−4.9 ± 5.3, *p* = *0.36*	--	−11.8 ± 6.9, *p* = *0.096*
Seizures in year prior to surgery	--	--	--	--	--	−7.7 ± 4.2, *p* = *0.076*	−15.7 ± 6.0, *p* = 0.014*
Seizures more than a year ago	--	--	--	--	--	4.5 ± 4.5, *p* = *0.33*	−4.1 ± 7.0, *p* = *0.56*
Dexamethasone presurgery	--	--	--	--	--	−2.8 ± 3.9, *p* = *0.48*	−3.5 ± 9.7, *p* = *0.72*
Dexamethasone at time of surgery	--	--	--	--	--	−7.3 ± 4.1, *p* = *0.081*	−14.2 ± 9.5, *p* = *0.15*
Levetiracetam	--	--	--	--	--	−7.9 ± 4.1, *p* = *0.059*	−15.4 ± 6.5, *p* = 0.024*
df	43	41	40	39	37	36	30
Residual sum of squares	3080	2554	2528	2476	2350	2111	1693
*R*^2^ (COD)	0.29	0.41	0.42	0.43	0.46	0.52	0.61
Adj. *R*^2^	0.23	0.33	0.32	0.32	0.32	0.37	0.39
ANOVA							
*F*	4.5	4.8	4.2	3.7	3.2	3.5	2.8
*p*	0.0041**	0.00081**	0.0016*	0.0026**	0.0050*	0.0022**	0.0069*

The dependent variable is tonic current. All independent variables are given in the first column. Coefficients for each model are given in pA along with SEs and with the associated *p* values given below in italics. Summary statistics for each model are given at the bottom of the table. For sex and hemisphere, coefficients were arbitrarily calculated such that they represent the predicted change in tonic amplitude if the patient was male or the tissue was taken from the left hemisphere, respectively.

### Analysis of tonic currents and patient parameters

By their nature, electrophysiological experiments in human cortical slices are less controlled than those conducted on rodent tissue. The tissue samples recorded from here come from patients with varying pathology, ages, and sex and were removed from a variety of cortical regions (Table 1). The surgical procedures and tissue transport may also lead to larger variability as compared with tissue from rodents. The measured tonic currents were therefore compared with some of these parameters to check for any systematic variation.

**Figure 8.**
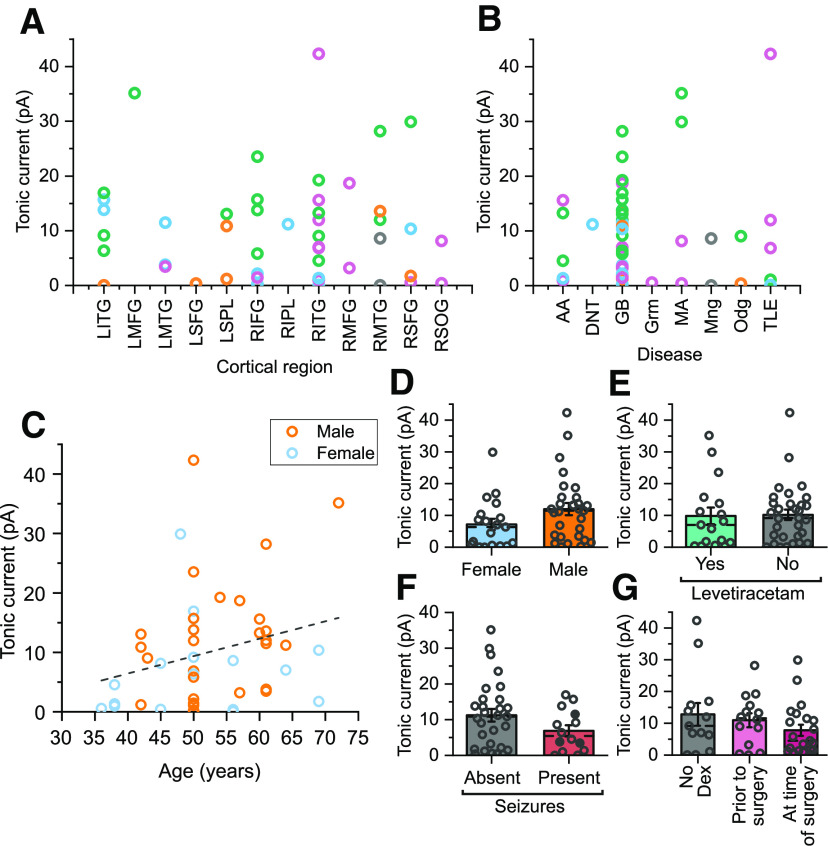
Relationships of tonic current amplitudes, firing clusters and patient populations. ***A***, Tonic current amplitudes are displayed by cortical region of the sample origin (LITG, left inferior temporal gyrus; LMFG, left middle frontal gyrus; LMTG, left middle temporal gyrus; LSFG, left superior frontal gyrus; LSPL, left superior parietal lobe; RIFG, right inferior frontal gyrus; RIPL, right inferior parietal lobe; RITG, right inferior temporal gyrus; RMFG, right middle frontal gyrus; RMTG, right middle temporal gyrus; RSFG, right superior frontal gyrus; RSOG, right superior occipital gyrus; one-way ANOVA: *F*_(36)_ = 0.83, *p* = 0.61). Individual cells are color coded according to the firing clusters identified in Figure 2. ***B***, Tonic currents are grouped by the pathology that resulted in the surgical removal of tissue (AA, anaplastic astrocytoma; DNT, dysembryoplastic neuroepithelial tumor; GB, glioblastoma; Grm, germinoma; MA, metastatic adenocarcinoma; Mng, meningioma; Odg, oligodendroglioma; TLE, temporal lobe epilepsy; one-way ANOVA: *F*_(40)_ = 0.85, *p* = 0.55). ***C***, Plot of the tonic current amplitudes against the age of the patients (orange, male; blue, female; Pearson's *r* = 0.26, slope = 0.29 pA/year, *t* = 1.81, *p* = 0.076). ***D***, Comparison of the amplitudes of currents measured from tissue taken from male and female patients (*t* test: *t*_(46)_ = 1.74, *p* = 0.089). ***E***, Comparison of currents measured from tissue taken from patients having received treatment with levetiracetam or not (*t* test: *t*_(46)_ = 0.12, *p* = 0.90). ***F***, Comparison of the tonic currents recorded in neurons obtained from tumor patients, broken down by whether their medical records indicate any history of seizures (*t* test: *t*_(41)_ = 1.18, *p* = 0.24). The three shaded data points represent tonic measures taken from patients who had a history of only focal seizures. All other data points were taken from those with a history of generalized seizures. ***G***, Comparison of currents by the dexamethasone (Dex) treatment received by the patient, broken down by whether it was administered only at the time of surgery or begun before that (6 d to three weeks; one-way ANOVA: *F*_(45)_ = 1.17, *p* = 0.32).

With regard to cortical region, the majority of the cells presented here with tonic current measures are from either the temporal (27 out of 48 cells), or frontal (15 out of 48 cells) lobes (Fig. 8*A*). No statistically significant difference in the tonic currents recorded from different cortical regions was observed (Fig. 8*A*), but this possibly reflects the relative lack of data on other cortical regions and therefore it would not be possible to rule out that such differences may be present between different regions of the human cortex. Similarly, no significant differences were observed in tonic currents recorded from patients with different pathologies (Fig. 8*B*). Although again, it should be noted that 28 of the cells were recorded from tissue taken from glioblastoma patients, with only a few taken from a variety of other oncology cases, and five from a single temporal lobe epilepsy case. It is also important to note that in the tumor cases, the tissue used for the study was mainly removed for the purpose of accessing the lesion rather than sourced directly from tumor itself, so it is possible that cells more proximal to the pathology may exhibit pronounced changes in their tonic currents.

A non-significant trend for increasing tonic current with age was observed (Fig. 8*C*), although it should be noted that the majority (77%) of the included cells were taken from patients in the age range of 50–65 (median of all included patients = 55 years, interquartile range = 46.5–61 years), with a relative lack of tissue from younger individuals. Additionally, a slight but non-significant trend toward lower tonic currents in cells taken from female patients was observed (Fig. 8*D*). A similar small and non-significant drop was observed for tumor patients with a history of seizures (Fig. 8*F*). Two of the more common drug treatments of patients included in the study, levetiracetam and dexamethasone, also did not appear to alter tonic current amplitudes. Levetiracetam, given both to treat seizures and prophylactically to some tumor patients, showed no significant difference in tonic currents (Fig. 8*E*). An analysis of dexamethasone treatment of the patients also showed no significant difference, although possibly a small non-significant decrease in tonic currents for patients who had first received dexamethasone at the time of the surgery (Fig. 8*G*)

The presence of such a large number of potential independent variables in a study using human tissue requires an analysis of which factors are relevant predictors of measured parameters. Multiple linear regression models were therefore generated to examine which variables were significant predictors of tonic inhibitory currents (Tables 2, 3). Models were generated separately using classification of the cells either by firing (Table 2), or axonal type (Table 3). For models classifying cells by firing, cluster 1 emerged as a consistent predictor of higher tonic currents (Table 2). The only other factor observed to be a predictor of higher tonic currents in these models was age. In the models with the highest adjusted *R*^2^ values (Table 2, models 2 and 3), tonic currents increased by 0.33 ± 0.16 pA/year. However, the relatively small age range of the patients from which tissue was obtained raises the need to test further age ranges to explore how far this trend goes throughout human lifespan. Similarly, for models classifying the cells by axonal type, rosehip cells and layer 2–3 innervating cells consistently predicted higher tonic current amplitudes (Table 3). Again, a small but significant increase in tonic current with age was observed with these models. Interestingly, in the model with the highest adjusted *R*^2^ value in this set (Table 3, model 13), which includes numerous clinical factors, a number of other variables were observed to predict changes in tonic currents. These included a significant decrease in tonic currents in patients with a history of seizures in the year before the surgery and in glioblastoma patients, and a significant increase in cells recorded from patients treated with levetiracetam. For the purposes of this modeling, patients with a history of seizures were separated based on whether seizures had been reported in the year preceding the surgery. This resulted in cells from two of the patients, who had no reported seizures for three to four years before the surgery from being separated from those with recent seizures. Cells from those two patients did not show a significant decrease in tonic currents (Table 3).

### Comparison of the responses of interneurons to GABA_A_R-selective modulators

**Figure 9.**
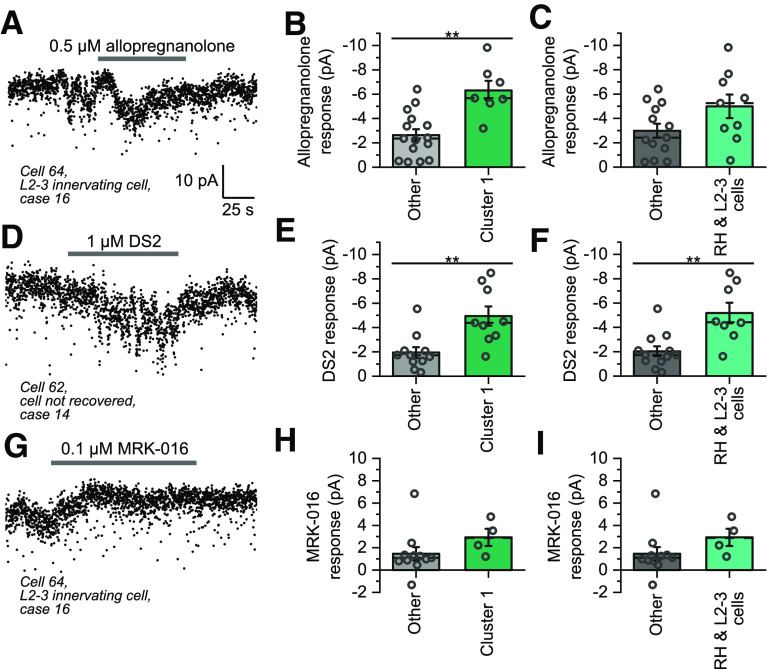
Responses of human interneurons to the GABA_A_R allosteric modulators allopregnanolone, DS2, and MRK-016. ***A***, Example current trace showing the response of a layer 1 interneuron to the bath application of 0.5 μm allopregnanolone. For panels ***A***, ***D***, ***G***, the traces were filtered before a 10-ms moving average of the current was taken every 100 ms, and all three modulators were applied in the presence of the 5 μm GABA. ***B***, Peak amplitudes of the current deflections induced by allopregnanolone application, divided by whether the recorded cells were in cluster 1 of the firing classification in Figure 2 (*t* test: *t*_(20)_ = 4.12, *p* = 0.00053, *n* = 22 cells). ***C***, Peak amplitudes of the current deflections induced by allopregnanolone application divided by whether the recorded cell had either a rosehip, or layer 2/3 innervating axon or not (*t* test: *t*_(20)_ = 1.89, *p* = 0.073). ***D***, Example current trace showing the response of an interneuron to bath application of 1 μm DS2. ***E***, Peak amplitudes of the current deflections induced by DS2 application, divided by whether the cell was in firing cluster 1 (*t* test: *t*_(18)_ = 3.51, *p* = 0.0025, *n* = 20 cells). ***F***, Peak amplitudes of the current deflections induced by DS2 application divided by whether the cell had either a rosehip or layer 2/3 innervating axon or not (*t* test: *t*_(18)_ = 3.71, *p* = 0.0016). ***H***, Peak amplitudes of the current deflections induced by application of MRK-016 divided by whether the cell was in firing cluster 1 (*t* test: *t*_(13)_ = −1.33, *p* = 0.21, *n* = 15 cells). ***I***, Peak amplitudes of the current deflections induced by treatment with MRK-016, divided by whether the cell had either a rosehip or layer 2/3 innervating axon or not (*t* test: *t*_(13)_ = −1.33, *p* = 0.21).

To probe the basis of the cell type differences in tonic inhibition, the responses of different interneurons to the GABA_A_R modulators allopregnanolone, DS2, and MRK-016 were compared. Because of limitation of tissue availability only one concentration could be tested for each, which was chosen deemed to be appropriate for tissue slices trying to find a balance between the slow penetration of the drug and selectivity of action. Higher concentrations may have resulted in larger effects, but potentially with less selectivity. Allopregnanolone is a neurosteroid that acts as a positive allosteric modulator at many GABA_A_R subtypes, but tends to have larger effects on receptors containing the δ subunit (Wohlfarth et al., 2002; Lu et al., 2020). The changes of currents induced by exposing the cells to allopregnanolone in the presence of exogenous GABA were significantly larger in cluster 1 cells (−6.3 vs −2.6 pA; Fig. 9*A*,*B*). However, when comparing by axonal classification, although rosehip and layer 2–3 innervating cells displayed larger responses on average, the difference between these two pooled groups and the rest of the tested cells was non-significant (Fig. 9*C*). This perhaps reflects diversity of the layer 2–3 innervating cells recorded from in this study, with the smallest allopregnanolone response in that group originating from a cell with firing that showed a regular pattern and falling into cluster 5 on the firing classification. Additionally, responses to the δ subunit selective modulator DS2 (Wafford et al., 2009; Jensen et al., 2013) were significantly larger when comparing by either firing (−4.4 vs −1.7 pA; Fig. 9*D*,*E*) or axonal classifications (Fig. 9*F*), indicating that rosehip and layer 2–3 cells or cells in firing cluster 1 are likely to have higher expression levels of the δ subunit. Conversely, the responses of most cells tested with the α5-preferring inverse agonist MRK-016 (Chambers et al., 2004; Atack et al., 2009; Atack, 2011) were small (Fig. 9*G–I*), and no significant difference between the groups was observed (Fig. 9*H*,*I*).

## Discussion

The results presented here show that the tonic currents of interneurons in layer 1 of human cortex vary widely, with cell type a key factor explaining some of this variation. We have delineated four distinct groups of interneurons based on their axonal features. Of the cells recorded in this study, tonic currents associated with the rosehip cells, and layer 2–3 innervating cells tended to be larger than those recorded from other cells. This finding is in some accordance with transcriptomic data showing that the rosehip cell is one of the few GABAergic cell types in human layer 1 that expresses the δ and α5 subunits of the GABA_A_R, which are commonly involved in tonic inhibition (Glykys and Mody, 2006, 2007). However, the same transcriptomic data also shows other LAMP5-expressing cells, including neurogliaform cells, express δ and/or α5, perhaps suggesting a further posttranscriptional control of tonic inhibition in layer 1. Interestingly, while the rosehip and layer 2–3 innervating cells also produced larger current deflections on application of the δ-selective allosteric modulator DS2, no significant difference was observed in their responses to the α5-selective inverse agonist MRK-016. Although we cannot completely rule out a contribution of receptors containing the α5 subunits to the tonic currents of these cells, and it is possible that higher concentrations of MRK-016 or more efficacious modulators of α5 may reveal one, overall the most parsimonious explanation is that a higher level of expression of the δ subunit is the primary cause of the elevated tonic currents observed in these cells. Intriguingly, these two cell groups defined by their very different axons also displayed remarkably similar firing properties, with many of the layer 2–3 innervating cells also displaying the high sag-ratio and irregularity characteristic of the rosehip cells. We also provide some evidence of tonic inhibition varying between patients, with regression modeling identifying age, seizures, levetiracetam treatment, and glioblastoma pathology as potential factors.

Among the labeled interneurons of layer 1, we have most frequently encountered the extensively described neurogliaform cells in humans (Kisvárday et al., 1990; Tamás et al., 2003; Oláh et al., 2007; Varga et al., 2015) and rodents (Hestrin and Armstrong, 1996; Overstreet-Wadiche and McBain, 2015; Schuman et al., 2019) and rosehip cells, which have been first reported in humans (Boldog et al., 2018). Although, on the basis of axonal distribution we observed “canopy”-like cells described in the mouse cortex (Schuman et al., 2019), with extensive straight lateral subpial axonal branches in our sample, we could not separate them from neurogliaform cells and “stalked-bouton cells” with an all or none criterion. Schuman et al. (2019) separated canopy cells and neurogliaform cells on the basis of only the latter expressing neuropeptide tyrosine (NPY) and being late-spiking; however, we observed that not all neurogliaform cells are late-spiking and transcriptomic data suggest that human neurogliaform cells do not express NPY (BRAIN Initiative Cell Census Network, 2020). We also encountered cells with descending axons to deeper layers as in rodents (Zhou and Hablitz, 1996; Jiang et al., 2013; Schuman et al., 2019), which also displayed higher tonic currents similar to those of the rosehip cells. In rodents two such cell types have been reported to have cell bodies in layer 1 with descending axons, expressing either α7 nicotinic acetylcholine receptors or vasoactive intestinal polypeptide (Schuman et al., 2019). However, we could not test for these molecular markers in our human sample, so whether such cell types have differences in their tonic GABA_A_R-mediated currents remains to be addressed in future studies.

The large tonic currents in rosehip cells have two implications. First, such a differential expression of tonic GABA_A_R currents could provide a target for the selective modulation of these cells within layer 1. The role of the specialized GABAergic rosehip cell is not fully understood in cortical circuitry, but Boldog et al. (2018) reported that they can inhibit calcium transients in the distal dendrites of pyramidal cells that they innervate. The high level of tonic inhibitory current could provide an avenue for the modulation of the excitability of rosehip cells in investigations of their roles. Second, the observation that a cell thus far only described in human tissue, that may be absent in rodents, exhibits large tonic currents may have implications for targeting of tonic inhibition in therapeutic strategies. This question is particularly relevant given recent success bringing neurosteroid based modulators to the clinic (Belelli et al., 2020). Although such drugs do not necessarily exhibit specificity for particular GABA_A_R subunit combinations (Lu et al., 2020), they certainly enhance tonic currents (Stell et al., 2003; Reddy and Estes, 2016), and their efficacy, particularly their sedating and anxiolytic properties, is diminished in δ−/− mice (Mihalek et al., 1999; Vicini et al., 2002; Stell et al., 2003; Sarkar et al., 2011; Leppä et al., 2016). Interestingly, neurosteroids have already shown promise in treating depressive disorders, with brexanolone (a formulation of allopregnanolone for intravenous infusion) receiving approval for postpartum depression (Paul et al., 2020) and SAGE-217 (an allopregnanolone analog) showing potential in phase 2 clinical trials for major depressive disorder (Gunduz-Bruce et al., 2019), albeit narrowly missing its primary endpoint in a phase 3 (Calistri and Boyle, 2019).

Additionally, albeit with tissue from a limited number of patients, we find evidence here that tonic inhibition may be altered by a number of clinical parameters, including age, seizures, levetiracetam, and type of cancer. Decreases in tonic inhibition (Pandit et al., 2013), or δ subunit expression (Peng et al., 2004; Rajasekaran et al., 2010; Drexel et al., 2013; Wan et al., 2014; Szyndler et al., 2018), have been observed in the hippocampi of rodent epilepsy models, particularly in the granule cells of the dentate gyrus, perhaps consistent with the decreased tonic inhibition observed in patients with seizures in this study. Interestingly, the trend toward a decrease in tonic currents in rosehip cells may suggest a cell type-specific effect of seizures. It is possible that such decreases in tonic inhibition of inhibitory interneurons might act as a homeostatic mechanism to compensate for general increases in excitability after seizures. However, demonstrating an increased excitability of inhibitory interneurons, as our results predict, will require further work. On the other hand, our observation that tonic current tends to increase with age would perhaps contrast with rodent studies showing decreases in tonic inhibition with age in both hippocampus and thalamus (Haberman et al., 2011; Richardson et al., 2013; McQuail et al., 2015). However, the lack of tonic current measures from younger patients in our study (youngest patient = 36 years) does raise the question of how far this trend to increasing tonic currents goes throughout human lifespan. The apparent effects of levetiracetam and glioblastoma are also unexpected. Whether the correlation with decreased tonic currents observed with levetiracetam reflects an effect of the drug or simply that its use predicts either a history of seizures or the potential for them in patients who have received it prophylactically will require further work.

The cell-type or pathology-related differences in tonic GABA_A_R-mediated inhibition found here may translate to other cortical layers or brain areas. Of particular interest will be the *PVALB*-expressing neurons in the deeper cortical layers as transcriptomic data indicate the expression of the δ subunit (BRAIN Initiative Cell Census Network, 2020) and may be under significant regulation by tonic GABA_A_R currents (Bryson et al., 2020). The layer 2–3 innervating cells identified in this study are also candidates for further characterization, particularly those that have firing patterns similar to those of the rosehip cells. Whether the similarities between the firing and tonic currents of these distinct cells represent a transcriptomic or developmental relationship, or just a convergence onto similar electrophysiological properties is an area to be explored. Knowledge of the synaptic targets of these layer 2–3 cells will also aid the elucidation of the roles of the large tonic currents reported here in the wider microcircuit. The rosehip cells are known to primarily output onto pyramidal cells (Boldog et al., 2018), suggesting that the observed tonic currents could perhaps have a role in restraining inhibitory output onto excitatory cells in the deeper cortical layers. Further study will also be needed to examine whether any of the differences between patient populations identified by modeling of the tonic currents presented here are reproducible in other layers of cortex, and particularly whether any of these effects extend to the cortical glutamatergic cells. One point of particular interest will be whether any potential seizure-induced changes in tonic inhibition in either the cells studied here or in other cells throughout the brain have any implications for the development of GABA_A_R modulators such as neurosteroids for use as anticonvulsants, particularly given the failures of ganaxolone and brexanolone in phase three trials for adult focal onset seizures and super-refractory status epilepticus respectively (Caperelli, 2016; Cox, 2017). In summary, this paper provides an insight into the heterogeneity of tonic currents in the diverse GABAergic interneuron population of the human neocortex, and some of the factors that affect these currents in neurooncological patients.
